# An amphibian species pushed out of Britain by a moving hybrid zone

**DOI:** 10.1111/mec.15285

**Published:** 2019-11-19

**Authors:** Jan W. Arntzen

**Affiliations:** ^1^ Naturalis Biodiversity Center Leiden The Netherlands

**Keywords:** *Bufo spinosus*, common toads, *Homo neanderthalensis*, introgression, leaky replacement, mid‐Holocene, species distribution models

## Abstract

Classical theory states that hybrid zones will be stable in troughs of low population density where dispersal is hampered. Yet, evidence for moving hybrid zones is mounting. One possible reason that moving zones have been underappreciated is that they may drive themselves into oblivion and with just the superseding species remaining, morphological and genetic signals of past species replacement may be difficult to appreciate. Using genetic data (32 diagnostic single nucleotide polymorphisms) from a clinal hybrid zone of the common toad (*Bufo bufo*) and the spined toad (*Bufo spinosus*) in France for comparison, alleles of the latter species were documented in common toads in the south of Great Britain, at frequencies in excess of 10%. Because long distance dispersal across the Channel is unlikely, the conclusion reached was that the continental toad hybrid zone which previously extended into Britain, moved southwards and extirpated *B. spinosus*. Species distribution models for the mid‐Holocene and the present support that climate has locally changed in favour of *B. bufo*. The system bears resemblance with the demise of *Homo neanderthalensis* and the rise of *Homo sapiens* and provides an example that some paleoanthropologists demanded in support of a hominin “leaky replacement” scenario. The toad example is informative just because surviving pure *B. spinosus* and an extant slowly moving interspecific hybrid zone are available for comparison.

## 1 INTRODUCTION

Hybrid zone studies have a prominent place in evolutionary research because they provide opportunities to study the mechanisms that keep species apart in the face of gene flow (Abbott et al., 2013; Ravinet et al., 2017). They can be seen as natural arenas where combinations of mutations from different species are functionally tested (Zieliński et al., 2019). Most recognized hybrid zones have come into existence upon the secondary contact of groups of organisms that are morphologically and genetically differentiated. Hybrid zones that are independent of the environment and in which the admixed offspring has lower fitness than the parents, are termed “tension zones”. These are maintained by an equilibrium between dispersal into the zone and selection against hybrids (Barton, 1979b; Barton & Gale, 1993; Key, 1968). Classical hybrid zone theory states that tension zones will be stable, or at least persistent, in troughs of low population density where dispersal is reduced. These troughs would be frequent and deep enough to resist large‐scale movement due to species fitness differences or asymmetry of hybridization (Barton, 1979b; Barton & Hewitt, 1985). The perspective of hybrid zone stability has, however, eroded under a flood of empirical data from three lines of evidence, namely (a) historical references and observations over time, (b) isolated populations surrounded by the superseding species (enclaves) and (c) alleles left behind by the receding species (genetic footprints) (Arntzen & Wallis, 1991; Buggs, 2007; Wielstra, 2019). One possible reason that moving hybrid zones have been underappreciated is that they may drive themselves into oblivion and with just the superseding species remaining, morphological and genetic signals of past species replacement may be difficult to appreciate. As Levin (2006) stated, finding the “ghost of hybridization past” can be elusive.

**Figure 1:**
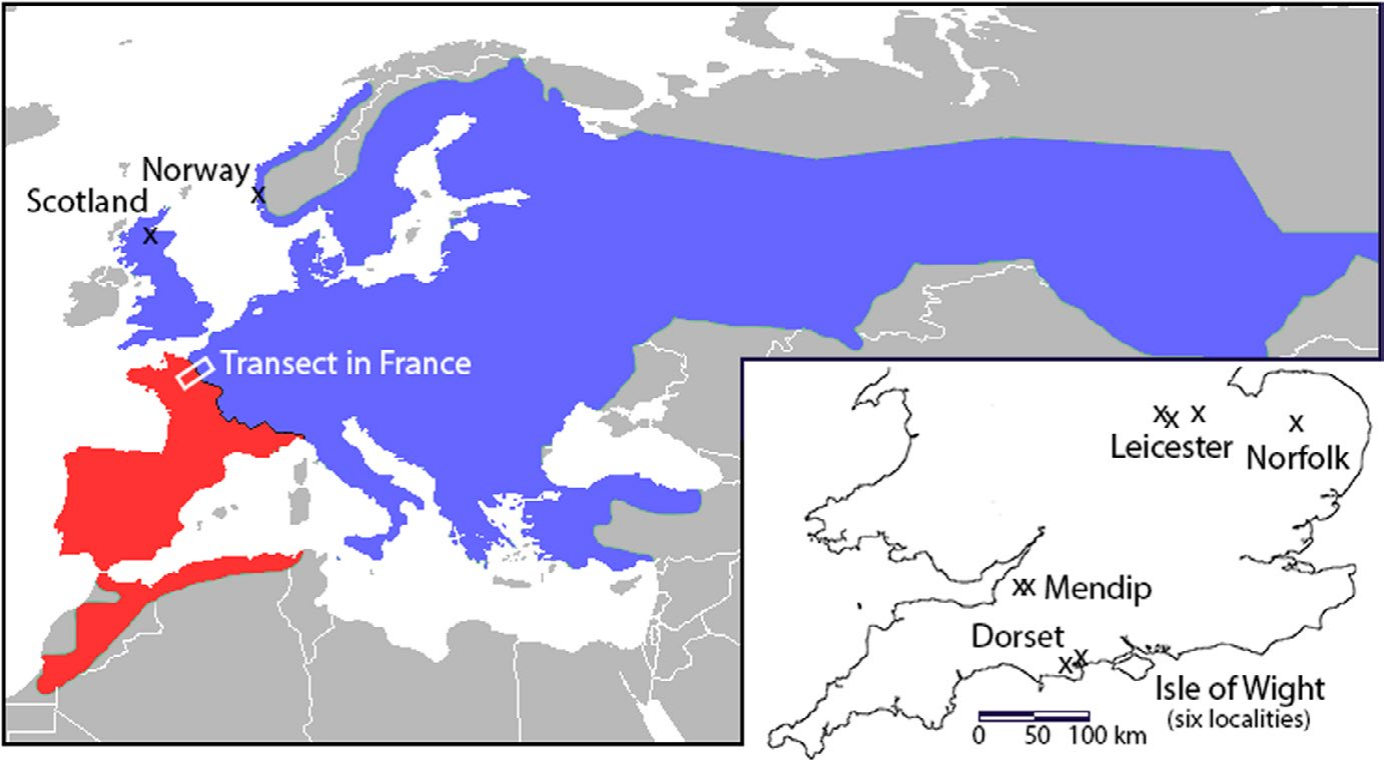
Distribution of the spined toad, *Bufo spinosus* (red) and the common toad, *Bufo bufo* (blue) after Agasyan et al. (2009) and Arntzen et al. (2018). Study localities are shown by x‐symbols. Inset: study localities in England. Note that *Bufo* toads are absent in Ireland and that their presence on the Isle of Man remains to be confirmed (NBNatlas, https://species.nbnatlas.org/species/NHMSYS0000080159)

This study follows the discovery and description of an amphibian hybrid zone across France that formed following species' northerly range expansions, starting with the Holocene glacial retreat (Arntzen, McAtear, Butôt, & Martínez‐Solano, 2018; Recuero et al., 2012; Trujillo, Gutiérrez‐Rodríguez, Arntzen, & Martínez‐Solano, 2017). The species involved are the common toad *Bufo bufo* to the northeast and the spined toad *Bufo spinosus* to the southwest of their mutual species border (Figure 1). Evidence is mounting for this hybrid zone to be moving along the Channel coast (Arntzen et al., 2016; van Riemsdijk, Butlin, Wielstra, & Arntzen, 2019). To test this hypothesis this study set out to investigate if—when the Channel had yet to be formed—the *Bufo* species contact might have extended into Great Britain.

## 2 MATERIALS AND METHODS

Tissue material was obtained as toe tips from adults and the tips of tailfins from larvae for 145 individuals at 15 localities in Great Britain (Table 1) and as extracted DNAs from 358 individuals from Dorset, United Kingdom (Coles, Reading, & Jehle, 2019) and 318 individuals at 21 localities in the southwest of Norway (Roth & Jehle, 2016). New material was collected in accordance with the UK's Wildlife and Countryside Act, 1981. Fluorescence‐based genotyping (Semagn, Babu, Hearne, & Olsen, 2014) was used in the Kompetitive Allele‐Specific PCR (KASP) genotyping system at the SNP (single nucleotide polymorphism) genotyping facility of the Institute of Biology, Leiden University. Primer design, PCR setup and data visualization followed Arntzen et al. (2016) and van Riemsdijk, Butlin et al. (2019). SNP's were selected on the basis of species diagnosticity. The data for Britain and Norway were analyzed jointly with published data that represent a *Bufo spinosus* to *Bufo bufo* hybrid zone in the northwest of France, along with reference populations from Spain, southern France and the Channel island Jersey (*B. spinosus*) and northern France and the Netherlands (*B. bufo*) (Arntzen et al., 2016; Arntzen, de Vries, Canestrelli, & Martínez‐Solano, 2017; Arntzen, Wilkinson, Butôt, & Martínez‐Solano, 2014; van Riemsdijk, Butlin, et al., 2019). The full panel of SNP's was studied with the exclusion of *c10orf2* and *klhl* that proved not diagnostic, *banp* that showed a widely displaced cline and *egflam* that was strongly out of Hardy‐Weinberg equilibrium (van Riemsdijk, Butlin, et al., 2019). Data for individuals with more than nine SNPs missing were discarded. For the remainder, missing data amounted to 2.8% (*N* = 974) for 31 nuclear markers and 0.1% (*N* = 1) for the one mitochondrial marker.

**Table 1:** Fifteen *Bufo bufo* populations studied in Great Britain with locality information and sample sizes

Region	Locality number	Eastern longitude	Northern latitude	Locality name	Sample size
Isle of Wight	B01	−1.245	50.623	Sandpit cops, Wroxall	8
	B02	−1.363	50.644	Village pound, Shorwell	6
	B03	−1.385	50.648	Blakes, Brighstone	8
	B04	−1.279	50.682	Standen house, Newport	8
	B05	−1.291	50.683	Marvel farm, Newport	8
	B06	−1.337	50.736	Rolls hill, Cowes	7
Dorset	B07_a	−2.109	50.677	Stoborough	4
	_b	−2.117	50.650	North of Purbeck hills	358
	B08	−1.891	50.876	Verwood	6
Mendip hills and Salisbury plains	B09_a	−2.702	51.280	Mendip	2
	_b	−2.677	51.262	Mendip Priddy pool	2
	_c	−2.651	51.261	Mendip Waldegrave pool	8
	B10	−2.093	51.239	Salisbury plains	7
Leicestershire and Norfolk	B11_a	−1.357	52.619	Cadeby quarry	12
	_b	−1.334	52.673	Bagworth park	19
	B12_a	−1.410	52.751	Fish pond	12
	_b	−1.299	52.758	Shepshed	11
	B13	−0.768	52.658	Braunston	2
	B14	0.884	52.483	Illington	8
Scotland	B15	−4.584	57.588	Loch na Crann, Highland	8

A two‐species distribution model was constructed by contrasting presence data for both species (Arntzen, Canestrelli, & Martínez‐Solano, 2019). It describes the contiguous ranges of *Bufo spinosus* and *B. bufo* across France and into Italy from environmental parameters and showed a good fit to the underlying data of 404 genetically investigated toad populations (AUC = 0.97 ± 0.007). The model was re‐estimated to only include climate variables (bio01–bio19) and then applied to the mid‐Holocene climate reconstructions of WorldClim v. 1.4 (Hijmans, Cameron, Parra, Jones, & Jarvis, 2005; the nine model codes are BC, CC, CN, HE, HG, IP, ME, MG and MR). The parameters bio14 and bio15 were excluded from the analysis because of their regular high discrepancy between data sets (Varela, Lima‐Ribeiro, & Terribile, 2015). In the absence of firm guidance of which climate reconstruction would be most appropriate to apply (Guevara, Morrone, & León‐Paniagua, 2019), distribution models were derived for all nine of them. For species distribution models for the last glacial maximum see Garcia‐Porta et al. (2012).

The genetic data were analyzed with genepop version 4.2 (Rousset, 2008) and HIest (Fitzpatrick, 2012). Statistical data analysis was with spss version 20 (IBM SPSS, 2016). Habitat models were visualized with ilwis 3.6 (ILWIS, 2009).

## 3 RESULTS

**Figure 2:**
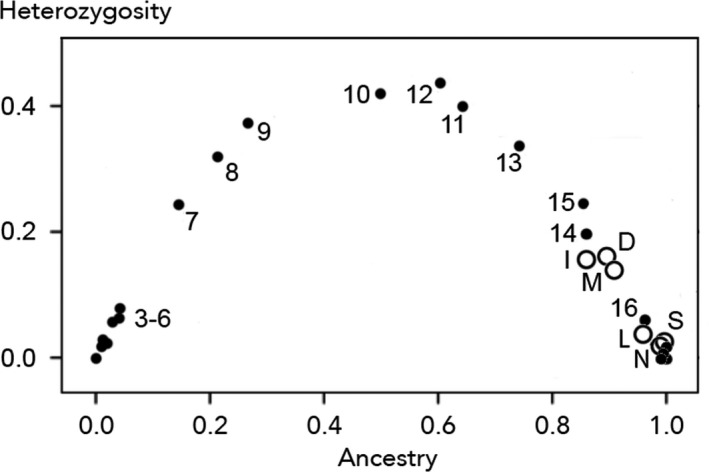
Bivariate plot for *Bufo* species ancestry and heterozygosity with HIest software (Fitzpatrick, 2012). Round symbols represent population averages, with solid dots for populations from continental Europe and Jersey and large open dots for British and Norwegian populations (I, Isle of Wight, population B01–B06 pooled; D, Dorset, B07 and B08; M, Mendip hills and Salisbury plains, B09 and B10; L, Leicestershire and Norfolk, B11–B14, S, Scotland, B15 and N, Norway, N01–N21). Populations 3–16 are genetically admixed and represent the 95% core of the *B. spinosus ‐ B. bufo* hybrid zone in the northwest of France. Approximately pure populations of *B. spinosus* and *B. bufo* are positioned in the lower left and lower right corners, respectively. For locality information and constituent data see Table 1, Supplementary Information S1, Arntzen et al. (2016) and van Riemsdijk, Butlin, et al. (2019)

Plotting species ancestry (*S*) versus population heterozygosity (*H*) produced a parabolic graph for the hybrid zone in France (Figure 2), with reference populations clustering in the lower left corner (*Bufo spinosus*, *S* < 0.025, *H* < 0.05) and in the lower right corner (*Bufo bufo*, *S* > 0.975, *H* < 0.05). Populations from mid‐England, Scotland and Norway fell into the lower right corner along with *B. bufo*, whereas populations from the south of Britain had positions akin to continental populations 14–16 that are situated at the northern section of the *B. spinosus* ‐ *B. bufo* hybrid zone transect in France. All British and Norwegian toads carried the mtDNA haplotype representative of *B. bufo*. At nuclear loci, the frequency of spinosus‐alleles (*F*_s_) was higher on the Isle of Wight (B01–06, 12.4% < *F*_s_ < 15.8%) than in the south of mainland Britain (B07–10, 7.9% < *F*_s_ < 11.2%) than to the north (B11–15 and N01–21, 0.4% < *F*_s_ < 5.8%) (Table 1). Genetic differentiation between these three population groups is significant (Fisher's method, *p* < .001 for the three pairwise comparisons). Allele frequencies for British and hybrid zone populations were to varying degrees correlated. The strongest signals were observed for the combination of southern British (B01–10) and northern hybrid zone populations (France, 12–16), with nine out of 15 pairwise comparisons statistically significant. Extremes in the marker spectrum are the loci *pigg* and *pomc*, with low respectively high frequencies of spinosus‐alleles in a *B. bufo* genetic background in either country. The population from Norway has a relatively high frequency of spinosus‐alleles (2.0% < *F*_s_ < 4.8%), which is almost entirely due to the contribution of the loci *aimp2* (*F*_s_ = 70.5%) and *med8* (*F*_s_ = 25.1%).

**Figure 3:**
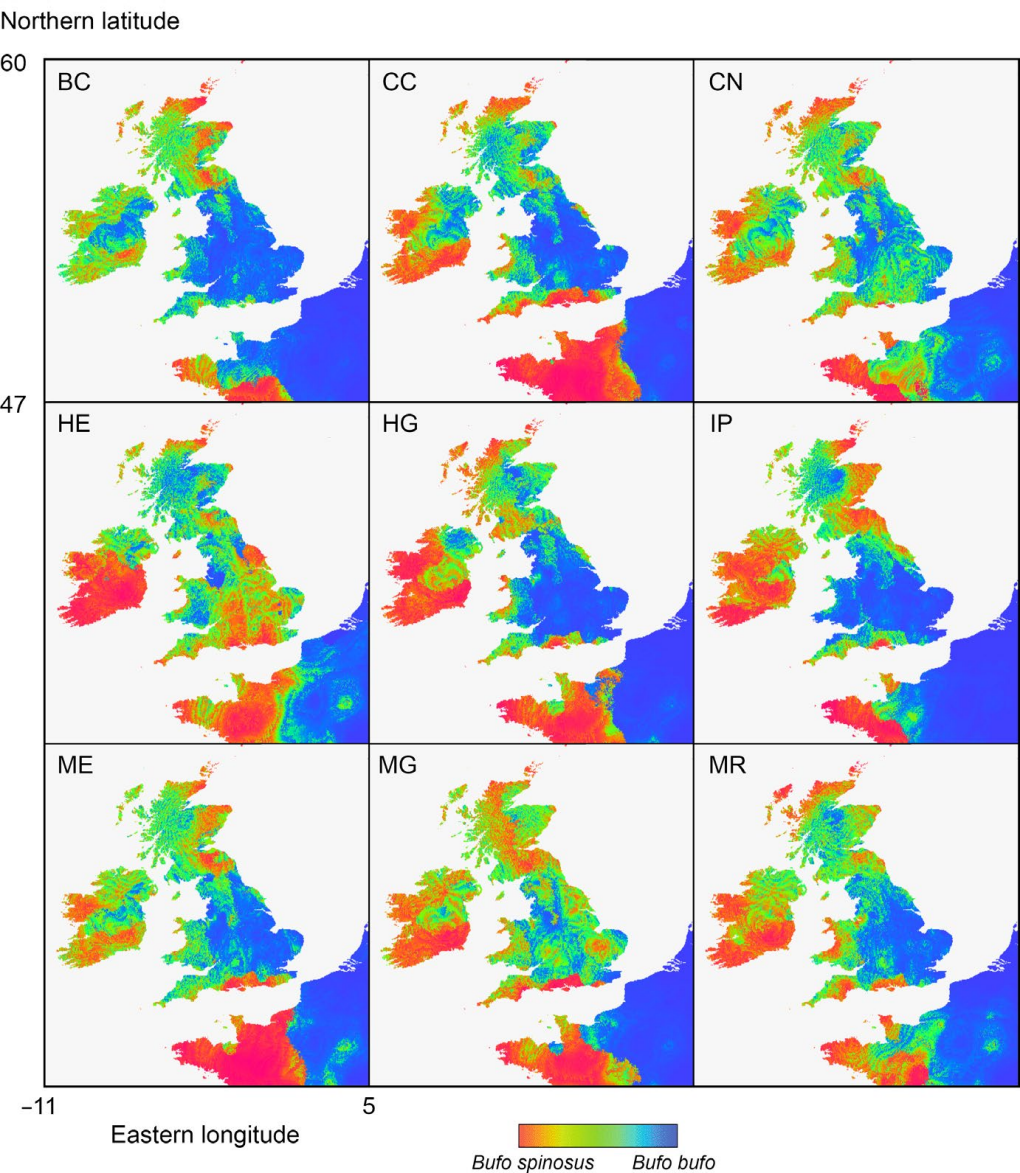
The northwest of Europe with two‐species distribution models derived for climatic conditions of the mid‐Holocene from nine different data sets (Hijmans et al., 2005). Advantageous conditions for *Bufo spinosus* and *B. bufo* are shown by red and blue colours, respectively, with intermediate colours indicating intermediate conditions (see colour legend). For species distribution models for the present and the last glacial maximum see Garcia‐Porta et al. (2012) and Arntzen et al. (2019)

The selected two‐species distribution model for climatic data is represented by the logistic equation *P*_b_ = (1/[1 + exp{0.0453bio01‐0.559bio02 + 1.423bio03 + 0.00844bio04‐0.0284bio06‐0.0237bio09‐51.278}]), in which *P*_b_ is the probability for the presence of *B. bufo* at the locality investigated, on a zero to unity scale. The model fit is AUC = 0.91 ± 0.015. Application of this model to the climatic conditions inferred for the mid‐Holocene of Great Britain yielded markedly different results for the nine climate models (Figure 3). Contrasting the northern (nearly) pure populations of *B. bufo* (B11–15, seven localities, *F*_s_ ≤ 5%) with the genetically admixed southern populations (B01–10, 13 localities, *F*_s_ > 10%) in the logistic equation presented above yielded a good model fit (AUC > 0.8) for the climate models CC (AUC = 0.96 ± 0.041), CN (AUC = 0.83 ± 0.094), HG (AUC = 0.88 ± 0.085), ME (AUC = 1 ± 0) and MR (AUC = 0.87 ± 0.084), and not for the four others (0.55 < AUC <0.69).

## 4 DISCUSSION

*Bufo bufo* populations in the south of England are genetically differentiated from more northern ones at a level that cannot be explained by local adaptation or genetic drift. The alleles underlying the genetic shift are typical for *Bufo spinosus* and their origin can be attributed to that species. A framework condition for hybridization to explain the data is that genetic isolation of the species is incomplete. Studies on their extant contact zone in France demonstrate that *B. bufo* and *B. spinosus* engage in successful hybridization (Arntzen et al., 2016; Trujillo et al., 2017). *Bufo* toads have some potential for oversea dispersal, as indicated by their presence on the Isle of Wight that is separated from the British mainland by the Solent sea strait of ≥2.4 km wide (present study, see also Roth and Jehle (2016) for populated islands in the Norwegian Holderland archipelago). However, recent very long distance dispersal, such as *B. spinosus* crossing the Channel where it is the narrowest (33 km) and traversing *B. bufo* territory at either side, or straight from Normandy to the Isle of Wight (96 km), is unlikely and a fitting scenario for the presence of *B. spinosus* genetic material in Britain will have a historical component.

### 4.1 Genetic and climatic support for a past enclave

Hybrid zone movement may result in substantial unidirectional introgression of selectively neutral material from the local to the advancing species, leaving a genetic footprint (Scribner & Avise, 1993; Wielstra et al., 2017). In France, a weak but statistically significant asymmetry of clines was observed in support of a genetic footprint and southward shift of the *B. spinosus – B. bufo* hybrid zone (van Riemsdijk, Butlin, et al., 2019). How long such movements leave genetic traces depends on the effective selection against hybrids, but may also vary depending on other factors related to hybrid zone movement (Currat, Ruedi, Petit, & Excoffier, 2008). It is reasonable to conclude upon the past presence of *B. spinosus* in the south of Great Britain from low (but not negligible) frequencies of spinosus‐alleles carried by *B. bufo*. The observation is best explained by a southward moving hybrid zone that replaced one species (*B. spinosus*) by the other (*B. bufo*) over a wide area.

Strengths of introgression may differ between genomic regions, depending on selection promoting or counteracting genetic exchange (Baack & Rieseberg, 2007; Roux, Tsagkogeorga, Bierne, & Galtier, 2013). Regions that introgress more easily than the genomic background are likely to contain adaptive variants, whereas the flow of the genes involved in reproductive isolation and genes linked to them is impeded and yet others, perhaps the majority, may be selectively neutral (Barton, 1979a; Piálek & Barton, 1997). The populations in Norway are almost completely devoid of spinosus‐alleles, except at the loci *aimp2* and *med8* (Table 2). Considering that genetic variation usually decreases towards a species' range edge, the standing genetic variation observed in Norway is high (Roth & Jehle, 2016). A pattern where alleles reappear at localities away from the admixture zone has been observed in European house mice (Teeter et al., 2009) and is suggestive of differential negative selection, stronger within the area of admixture than away from it. The rare alleles may, however, also reflect a different (i.e., northeastern) post‐glacial colonization route for Scandinavia from a source carrying these variants, versus a more southern, homozygous origin for France and Great Britain. To resolve if the *B. spinosus* range extended into Scandinavia requires a wider phylogeographic study.

**Table 2:** Strength of correlations for the frequencies of alleles typical for *Bufo spinosus* (*F*_s_) over 31 nuclear SNP markers between (a) 14 common toad populations that represent the *B. spinosus* ‐ *Bufo bufo* hybrid zone in the northwest of France (Arntzen et al., 2016; van Riemsdijk, Arntzen, et al., 2019; van Riemsdijk, Butlin, et al., 2019) and *B. bufo* from (b) Great Britain, pooled in five regions arranged from south to north and (c) Norway

(a) France					(b) Great Britain					(c) Norway
Locality number and name	Distance from hybrid zone centre (km)	Sample size (*N*)	Frequency *B. spinosus* alleles (*F*_s_, %)		Isle of Wight (B01–06)	Dorset (B07−08)	Mendip hills and Salisbury plains (B09–10)	Leicestershire and Norfolk (B11–14)	Scotland (B15)	Hordaland archipelago (N01–21)
				*N*	45	368	19	64	8	318
				A	14.6	11.2	10.1	4.9	1.4	3.1
				R	12.4–15.8	7.9–11.2	9.9–10.5	0.4–5.8		2.0–4.8
				A#	12.3	11.1	9.5	2.7	0.9	0.0
				R#	10.2–13.7	7.8–11.1	8.8–10.7	0.4–3.3		0.0–0.2
3. Les Tesnières, Durtal	−135.0	8	95.7		**−0.15**	**−0.15**	**−0.08**	**0.23**	**−0.17**	**0.00**
4. Carrefour du Poteau, Jublains	−10.7	10	97.0		0.55^**^	**0.12**	**0.28**	**0.09**	**−0.18**	**0.04**
5. Les Fontaines, Pré‐en‐Pail	−74.5	8	95.8		**0.16**	**−0.14**	**−0.07**	**−0.07**	**−0.12**	**0.06**
6. Forêt de Multonne, Mont des Avaloirs	−69.9	12	95.8		**0.02**	**−0.24**	**−0.02**	**−0.03**	**−0.29**	**−0.09**
7. Montmean, Bazoches‐sur‐Hoëne	−23.2	19	85.5		**0.04**	**−0.08**	**0.06**	**−0.10**	**−0.18**	**−0.13**
8. La Couvendière, Mortagne au Perche	−16.3	20	78.8		**0.00**	**−0.16**	**0.05**	**0.23**	**−0.06**	**0.01**
9. La Rosière, Forêt du Perche et de la Trappe	−11.9	20	73.4		**0.22**	**−0.17**	**0.01**	**−0.15**	**−0.16**	**0.10**
10. Beaulieu	0.0	20	50.5		0.43^*^	**0.29**	**0.23**	**0.26**	0.41^*^	**0.08**
11. Chateau des Bois Francs	7.7	10	36.3		**0.35**	**0.11**	**0.30**	**0.08**	**−0.03**	**0.04**
12. Le Cottin	7.2	18	39.9		0.62^***^	**0.26**	0.45^*^	**0.29**	**0.04**	**0.07**
13. Les Quatre Vouges	24.0	20	26.2		**0.18**	**0.15**	**0.17**	**0.33**	0.40^*^	**0.04**
14. Mouettes	51.3	10	14.7		0.41^*^	0.40^*^	**0.24**	**0.18**	**0.10**	**0.00**
15. Mare du Bois, La Houssaye	52.0	12	14.8		0.67^***^	0.48^**^	0.48^**^	**0.34**	**0.28**	**0.20**
16. Les Puits du Sarrasin, Erloy	264.1	20	4.2		0.36^*^	**0.29**	0.44^*^	0.57^**^	**0.33**	**0.08**

Note

The correlation coefficient applied is that of Spearman (*r*_s_), with values and significances shown as bold (*p < .05, **p < .01, ***p < .001 and no mark *p *> .05, not significant).

Abbreviations: A, average *F*_s_; R, *F*_s_ range; #, markers *aimp2* and *med8* excluded.

The frequencies of introgressed alleles in Britain and France are correlated across loci (Table 2). This result could be explained by the existence of a single, continuous hybrid zone for a long time, so that gene flow within species generated associations of allele frequencies between Britain and France. However, both sections of the hybrid zone became disconnected at c. 7.5 kya (see below) and ever since, British and French population groups followed independent evolutionary trajectories. Given the potential for differentiation, the observed correlations indicate that the introgression processes were similar in both countries, suggesting that a large proportion of the studied loci are affected by some kind of selection. Genetic markers that show consistent patterns of restricted introgression across multiple, spatially well separated transects may mark genome regions that are components of a universal species boundary (Larson, White, Ross, & Harrison, 2014; Teeter et al., 2009; van Riemsdijk, Arntzen, et al., 2019). A prime candidate for negative selection in the present experiment is the locus *pigg*, with low and zero frequencies of spinosus‐alleles in a *B. bufo* genetic background at both sides of the Channel.

Although the genetic footprint of *B. spinosus* in the south of Britain is unequivocal, it does not represent a full‐blown enclave because it concerns the residual presence of *B. spinosus*, which is interpreted as an enclave that has eroded, or a semi‐enclave, because the *B. spinosus* stronghold was presumably never surrounded by *B. bufo*, but rather sandwiched between this counterpart species and the Channel. An alternative scenario is that just hybrid populations were present in the south of Britain. However, to explain the large area of admixture the zone should have been wide, which is at odds with documented dispersal and selection estimates and the observed hybrid zone width of 50 km on the continent (Arntzen et al., 2019, 2016; van Riemsdijk, Arntzen, et al., 2019; van Riemsdijk, Butlin, et al., 2019). A long and winding hybrid zone is also unlikely because the position of the contiguous species border appears to be determined by landscape elements such as mountains and major rivers (Arntzen et al., 2018, 2019).

For Great Britain, species distribution models for the past and present suggest that climatic conditions improved for *B. bufo* relative to *B. spinosus* (Arntzen et al., 2019 and Figure 3), thereby supporting a scenario of species replacement under climate change. Extant populations with traces of ancient hybridization are associated with mid‐Holocene climatic conditions preferred by *B. spinosus*, with good fit for the majority of models. For what is currently known, the last British resort for *B. spinosus* may have been the Isle of Wight where it was sheltered from invading *B. bufo* by the Solent sea strait. However, the mid‐Holocene climate models raise the question if not a *B. spinosus* genetic footprint would also be found in other areas with past favourable climatic conditions including Cornwall, the coastal zone of Wales and parts of Scotland. Such observations might be aligned with Oceanic climate preferences and with the "Celtic fringe" pattern of intraspecific lineage replacement that has been postulated for several small mammals (Brace et al., 2016; Kotlík et al., 2006; Kotlík, Marková, Konczal, Babik, & Searle, 2018; Piertney et al., 2005; Searle et al., 2009). Of particular interest in this respect are the records for peripheral and isolated toad populations on Lundy Island and the Isles of Scilly and the possible presence of toads on the Isle of Man (NBNatlas, 2019).

### 4.2 Historical biogeography

The common toad *B. bufo* and the spined toad *B. spinosus* are morphologically similar yet genetically deeply differentiated species, with a most recent common ancestor dating back to the Miocene (Ehl, Vences, & Veith, 2019; Recuero et al., 2012). Their current spatial contact originated in the Holocene, through range expansion from glacial refugia in Spain and the south of France (*B. spinosus*) and the northern Balkans or beyond (*B. bufo*) (Arntzen et al., 2017). A relatively short period of climate warming during the Older Dryas (the Bøling‐Allerød period beginning at 14.7 kya) provided a first opportunity for northward expansion in many vertebrate species (Montgomery, Provan, McCabe, & Yalden, 2014). *Bufo* toads may not have been amongst these early range expanders, or not have been dispersing fast enough to reach Ireland before it became disconnected from the European continent at c. 15 kya (Montgomery & Provan, 2014). Range expansions may have temporarily stagnated during the Younger Dryas (12.9–11.7 kya), the last cold event of the Pleistocene preceding the final warming at the beginning of the Holocene (Montgomery et al., 2014). However, both toad species did make it into Britain, traversing the now inundated “Doggerland” landmass. Under a warming climate and a rising sea level the former “Channel river” (Antoine et al., 2003) enlarged to become the Channel. Consequently, the later a species' expanding range hit upon the Channel coast, the more northerly the overland route to Britain was located. The extant but eroded enclave indicates that *B. spinosus* was the first species to arrive. A spatial argument underpinning this notion is that the distance from the nearest glacial refugium is shorter for *B. spinosus* (c. 900 km) than for *B. bufo* (c. 1,500 km) (Arntzen et al., 2017). Currently no data are available that compare locomotion speed, but given that they are morphologically similar species, differences may be small. On its way north *B. spinosus* reached Normandy and the Cotentin peninsula 8 kya the latest, because the species made it into Jersey, which was the last of the Channel Islands to be separated from the European continent (Johnston, 1981). Entering Britain implies that *B. spinosus* expanded its range through the Low Countries, reaching as far north as the then adjoined Thames, Rhine and Meuse estuaries (Coles, 2000). For either species, the latest opportunity to make it into Britain was at 7.5 kya (Yalden, 1999), which also provides a minimum age for the *B. spinosus* ‐ *B. bufo* species interaction. Given its current position the hybrid zone may have then travelled with an estimated speed of 40 m per year. Hybrid zone movement may have been slower if more time was available, or it may have been higher prior to reaching terrain that *B. bufo* finds more difficult to cross, such as the Collines de Normandie, a range of hills that appears to act as a barrier to further southward displacement (Arntzen et al., 2016).

Other anuran species native to the British Isles are the common frog, *Rana temporaria,* and the natterjack toad, *Epidalia calamita*, which may have had a glacial refugium in or near the southwest of Ireland, on what was then dry land (Forbes, 1846; Rowe, Harris, & Beebee, 2006; Teacher, Garner, & Nichols, 2009). A similar scenario for *Bufo* toads is unsupported because either species is absent from Ireland.

### 4.3 Genetic footprints, and parallels with the demise of *Homo neanderthalensis*

The frequency of spinosus‐alleles in Britain decreases from south to north, with most of the decrease (from c. 15% to 5%) taking place over a distance of 200 km, from the South of England to the Midlands. Yet, data are currently insufficient to determine the extent of the past *B. spinosus* enclave in the south of Britain. This not just reflects limited spatial and genetic sampling, but also population genetic processes including drift, selection and neutral diffusion that can reduce, amplify or distort spatial genetic signals, as well as habitat dependent demographic parameters such as reproductive success and the propensity for dispersal (see Excoffier, Foll, & Petit, 2009; Harrison & Larson, 2014 for reviews). Whether the stronghold of *B. spinosus* in the south of Britain was large or small, a moving hybrid zone may have reduced its size and once dispersal of *B. spinosus* into the hybrid zone stalled the stabilizing equilibrium became disrupted and its fate was sealed. No *B. spinosus* mtDNA haplotypes could be found in Great Britain or Norway and it is interesting to note that this parallels the absence of records for *Homo neanderthalensis* mtDNA in present day *Homo sapiens* (Currat & Excoffier, 2004).

Hypothetical larger dispersal and long‐distance dispersal would widen a hybrid zone and disturb the typically clinal character‐state transitions (Barton & Hewitt, 1985; Ibrahim, Nichols, & Hewitt, 1996). An erratic, large‐scale pattern appears to describe the spatial‐genetic interactions of the lineages of anatomically modern humans and for example, Neanderthals, with frequent interbreeding when hominin species' ranges overlapped (Petr, Pääbo, Kelso, & Vernot, 2019; Slon et al., 2018; Vernot & Akey, 2014). The inferred species transition that was labelled as “leaky replacement” (Pääbo, 2015; see also Gibbons, 2011), parallels that of a moving hybrid zone leaving a genetic footprint. One difference may be that leaky replacement implies that introgressed alleles increase the fitness of the invading species (as in adaptive introgression: Hedrick, 2013; Pardo‐Diaz et al., 2012; Racimo, Sankararaman, Nielsen, & Huerta‐Sánchez, 2015), but the consulted literature is not explicit at this point. In spite of a long history of investigation, considerable debate revolves around whether Neanderthals became extinct because of climate change or competition with anatomically modern humans (Banks et al., 2008; Benito et al., 2017; Gilpin, Feldman, & Aoki, 2016; Kolodny & Feldman, 2017; Melchionna et al., 2018) and the degree to which they hybridized (Currat & Excoffier, 2004; Neves & Serva, 2012; Villanea & Schraiber, 2019).

In a provocative commentary on the leaky replacement scenario, Varki (2016) wonders “Why are there no persisting hybrids of humans with Denisovans, Neanderthals, or anyone else?” and “Why did hybrid species not persist, at least at the geographical extremes of BMH (behaviorally modern humans) expansion?”, and states “I cannot find any other example wherein a single (sub)species from one geographic origin completely replaced all extant cross‐fertile (sub)species in every planetary location, with limited introgression of functional genetic material from replaced taxa, and leaving no hybrid species. Typically, one instead finds multiple cross‐fertile (sub)species with hybrid zones in between.” These concerns can be addressed as follows. First, hybrid zones are frequently formed between taxa that are genetically differentiated and have low dispersal, but are less likely to form in restricted geographic spaces in highly dispersive and recently diverged taxa (see McEntee, Burleigh, & Singhal, 2018 for a review). Second, even inside well‐developed hybrid zones early generation hybrids may be rare, for example due to assortative mating, or to habitat segregation as in mosaic hybrid zones in which taxa map onto patches of interdigitated habitats. Third, the author seems to refer to stable hybrid zones, therewith ignoring that hybrid zones may be moving and ephemeral (Buggs, 2007; Wielstra, 2019). Fourth, uncovering traces of introgression, as in case of the *H. sapiens* ‐ *H. neanderthalensis* interaction, requires extensive data that may be available in hominin paleogenomics (Fu et al., 2016; Racimo et al., 2015; Sankararaman, Mallick, Patterson, & Reich, 2016) but not in most non‐model organisms. Finally, to brush the leaky replacement scenario aside as “human exceptionalism” (Varki, 2016– a qualification that made it into the textbook of Coolidge & Wynn, 2018) for lack of parallel examples from other organisms seems a little awkward. Proof of principle as here presented for European *Bufo* toads should satisfy most investigators. The toad example is convincing exactly because the species replacement is incomplete, with surviving pure *B. spinosus* and a slowly moving interspecific hybrid zone available for comparison. Disregarding temporal inequalities, *B. spinosus* temporarily survived in the very south of Britain just as Neanderthal tribes may have persisted for longest at the southernmost fringe of continental Europe, i.e., the Gibraltar peninsula (Carrión et al., 2018; Jennings, Finlayson, Fa, & Finlayson, 2011).

## AUTHOR CONTRIBUTIONS

The project was designed and carried out by JWA, who also prepared the manuscript.

## Data Availability

Newly obtained SNP‐data are provided in Supplementary Material S1
