# Supplementary material for: An amphibian species pushed out of Britain by a moving hybrid zone
Source: Mol Ecol. 2019 Nov 19;28(23):5145–54. doi: 10.1111/mec.15285 (PMC6900066; doi:10.1111/mec.15285)
Supplement: Supplementary file 1 [file MEC-28-5145-s001.docx]

**Supplemental Information to the article**

**‘An amphibian species pushed out of Britain by a moving hybrid zone’**

by Jan W. Arntzen

Naturalis Biodiversity Centre, P. O. Box 2300 RA Leiden, The Netherlands

**Supplemental Information I**

SNP-data for newly studied *Bufo* toad populations from Great Britain and Norway.

Marker names

aimp2, ankrd26, arhgap32, b4galt7, bdnf, brca2, c21orf2, c8b, cwc22, dbr1, eif5b, exon1, fam129b, gatsl2, gins3, lrrc23, med8, mesdc2, mrpl144, mthfr, ntrk2, pdgfrl, pigg, pomc, psmg3, rag1, rfc1, rnpepl1, rpl3, sart3, ttc37

[Locality_number_and_name/sample, 1 = bufo, 2 = spinosus and -9 = missing data]

B01_Sandpit_Cops_Wroxall/5074, 22 11 12 11 12 22 11 11 11 11 11 11 11 11 11 11 11 11 11 11 11 11 11 12 12 11 11 11 11 11 12

B01_Sandpit_Cops_Wroxall/5067, 22 11 11 11 11 12 -9 11 11 12 11 -9 11 11 11 11 12 11 11 11 11 11 11 22 12 11 11 11 11 11 11

B01_Sandpit_Cops_Wroxall/5068, 12 11 12 11 22 22 11 11 11 12 11 11 11 11 11 11 11 11 12 11 11 11 11 22 11 11 11 11 11 11 11

B01_Sandpit_Cops_Wroxall/5069, 22 11 11 12 11 22 11 11 11 11 11 11 11 11 11 11 22 11 11 11 11 11 11 22 12 11 11 12 11 11 11

B01_Sandpit_Cops_Wroxall/5070, 22 11 12 11 11 12 11 11 11 11 11 11 11 11 11 11 11 11 11 11 12 11 11 22 11 12 11 11 11 11 11

B01_Sandpit_Cops_Wroxall/5071, 22 11 12 11 11 12 11 11 11 11 11 11 11 11 11 11 11 11 11 11 11 11 11 22 11 12 11 11 11 11 11

B01_Sandpit_Cops_Wroxall/5072, 22 11 12 11 22 12 12 11 11 11 11 11 11 11 11 11 11 11 12 11 12 11 11 22 11 12 11 11 11 11 11

B01_Sandpit_Cops_Wroxall/5073, 22 11 12 12 12 11 11 11 11 11 11 11 12 11 11 11 12 11 11 11 11 11 11 22 11 11 11 11 11 11 11

B02_Village_Pound_Shorwell/5092, 22 11 12 11 12 12 12 11 11 12 11 11 11 11 11 11 11 11 11 11 11 11 11 22 11 11 11 11 11 11 22

B02_Village_Pound_Shorwell/5089, 12 11 11 11 11 12 11 11 11 12 11 11 11 11 11 12 11 11 12 11 11 11 11 22 11 11 11 11 11 11 12

B02_Village_Pound_Shorwell/5090, 12 12 12 11 12 12 11 11 11 12 11 11 11 11 11 11 11 11 22 11 11 11 11 22 11 11 11 11 11 11 12

B02_Village_Pound_Shorwell/5091, 12 12 12 11 11 11 11 11 -9 12 11 11 11 11 11 11 11 11 12 11 11 11 11 22 11 11 11 11 11 11 12

B02_Village_Pound_Shorwell/5093, 12 11 11 11 11 12 11 11 11 12 11 11 11 11 11 -9 11 11 22 11 11 11 11 22 11 11 11 -9 11 11 12

B02_Village_Pound_Shorwell/5094, 12 11 11 11 11 12 11 11 11 12 11 11 11 11 11 11 11 11 12 11 11 11 11 22 11 11 11 11 11 11 11

B03_Blakes_Brighstone/5096, -9 22 12 11 11 11 11 11 11 11 11 11 11 11 11 11 11 11 12 11 11 11 11 22 12 11 11 11 11 11 12

B03_Blakes_Brighstone/5097, 22 12 11 11 12 12 11 11 11 11 11 11 11 11 11 11 11 -9 22 11 12 11 11 22 -9 11 11 11 11 11 12

B03_Blakes_Brighstone/5100, 12 11 11 11 12 11 12 11 11 11 11 11 11 11 11 11 11 11 22 11 11 11 11 22 11 11 11 12 12 11 12

B03_Blakes_Brighstone/5101, 12 11 11 12 22 11 11 11 11 22 11 11 11 11 11 11 11 12 11 11 11 11 11 22 11 11 11 11 11 11 12

B03_Blakes_Brighstone/5102, 12 11 11 11 12 11 11 11 11 11 11 11 11 11 11 11 12 11 12 11 11 11 11 22 12 11 11 12 12 11 12

B03_Blakes_Brighstone/5095, 22 12 11 11 12 11 11 11 11 11 11 11 11 11 11 11 11 11 12 11 12 11 11 22 11 11 11 11 11 11 11

B03_Blakes_Brighstone/5098, 22 11 11 11 22 11 11 11 11 12 11 11 11 -9 11 12 11 11 22 11 12 11 11 22 12 11 11 11 11 11 11

B03_Blakes_Brighstone/5099, 22 11 11 11 12 11 11 11 11 11 11 11 11 11 11 -9 11 11 11 11 12 11 11 -9 11 11 11 12 11 11 11

B04_Standen_House_Newport/5049, 22 22 11 11 11 12 11 11 11 11 11 12 11 11 11 11 11 11 11 11 -9 11 11 22 11 11 11 12 11 11 12

B04_Standen_House_Newport/5051, 12 11 11 11 11 11 11 11 11 12 11 11 11 11 11 11 11 11 11 11 11 11 11 -9 11 11 11 12 22 11 12

B04_Standen_House_Newport/5053, 12 11 12 12 12 22 11 11 11 12 11 11 11 11 11 11 11 11 11 11 11 11 11 22 11 11 11 11 11 11 12

B04_Standen_House_Newport/5050, 22 11 12 11 11 12 11 11 11 11 11 11 11 11 11 11 12 11 11 11 11 11 11 -9 -9 11 11 11 12 11 11

B04_Standen_House_Newport/5052, 22 11 12 11 11 11 11 11 11 11 11 11 11 11 11 11 11 11 11 11 11 11 11 22 11 11 11 22 12 11 11

B04_Standen_House_Newport/5054, 12 -9 11 11 12 12 11 11 11 11 11 11 11 11 11 11 12 11 12 11 11 11 11 22 11 11 11 11 12 11 11

B04_Standen_House_Newport/5055, 12 11 11 12 11 12 11 11 11 11 11 11 11 11 11 11 12 12 11 11 11 11 11 -9 11 11 11 12 11 11 11

B04_Standen_House_Newport/5056, 12 11 12 12 12 11 11 11 11 11 11 11 11 11 11 11 11 11 11 11 12 11 11 -9 11 11 11 12 11 11 11

B05_Marvel_Farm_Newport/5033, 22 11 11 11 11 12 -9 11 11 11 11 11 11 11 11 11 11 11 12 11 11 11 11 22 11 11 11 11 11 11 11

B05_Marvel_Farm_Newport/5034, 12 11 11 11 12 11 11 11 11 11 11 11 11 11 11 11 11 12 11 11 11 11 11 22 12 12 11 12 12 11 11

B05_Marvel_Farm_Newport/5035, 11 12 12 11 11 11 11 11 11 11 11 11 11 11 11 11 11 11 11 11 11 11 11 12 11 22 11 12 12 11 11

B05_Marvel_Farm_Newport/5036, 11 11 12 12 12 12 11 11 11 11 11 11 11 11 11 11 12 11 11 11 11 11 11 22 11 11 11 11 12 11 11

B05_Marvel_Farm_Newport/5037, 12 11 11 11 11 11 11 11 11 11 11 11 11 11 11 11 11 11 11 11 11 11 11 22 11 11 11 11 11 11 11

B05_Marvel_Farm_Newport/5038, 12 11 12 12 12 12 11 11 11 11 11 11 11 11 11 11 22 11 11 11 11 12 11 22 11 11 11 12 12 11 11

B05_Marvel_Farm_Newport/5039, 22 11 11 11 11 12 11 11 12 11 11 11 11 11 11 12 11 11 22 11 11 11 11 22 12 11 11 12 12 11 11

B05_Marvel_Farm_Newport/5040, 22 11 11 12 22 11 12 11 11 11 11 11 12 11 11 12 12 11 11 11 11 11 11 22 12 11 11 12 22 11 11

B06_Rolls_Hill_Cowes/5011, 22 11 12 11 12 12 11 11 11 11 11 11 11 11 11 11 11 11 12 11 11 11 11 -9 12 11 11 11 12 11 12

B06_Rolls_Hill_Cowes/5012, 22 11 22 11 22 11 11 11 11 11 11 11 11 11 11 11 11 11 12 11 11 11 11 22 11 11 11 11 11 11 12

B06_Rolls_Hill_Cowes/5013, 22 11 12 11 11 12 12 11 11 12 11 11 11 11 11 11 12 11 11 11 11 11 11 22 11 11 11 11 12 11 12

B06_Rolls_Hill_Cowes/5016, 22 11 -9 11 22 11 11 11 11 11 11 11 11 11 11 11 11 11 11 11 11 11 11 22 11 11 11 11 12 11 12

B06_Rolls_Hill_Cowes/5017, 22 12 11 11 -9 12 11 11 11 11 11 -9 11 11 11 11 12 11 11 11 11 11 11 22 12 11 11 11 12 11 12

B06_Rolls_Hill_Cowes/5014, 22 11 12 11 22 11 11 11 11 11 11 11 11 11 11 11 22 11 11 11 11 11 11 22 -9 11 11 11 12 11 11

B06_Rolls_Hill_Cowes/5015, 22 12 12 11 12 12 11 11 11 11 11 11 11 11 11 11 11 11 11 11 11 11 11 22 11 11 11 11 12 11 11

B07_Stoborough/4315, 11 11 11 11 12 11 11 11 11 11 11 11 11 12 12 11 12 11 11 11 12 11 11 12 -9 11 11 11 11 11 -9

B07_Stoborough/4314, -9 11 11 11 11 11 12 11 11 11 11 12 22 11 12 11 11 11 11 -9 11 11 11 12 12 11 11 11 12 11 12

B07_Stoborough/4311, -9 22 11 11 11 11 11 11 11 11 11 11 11 11 22 11 11 11 11 11 11 11 11 11 11 11 11 11 12 11 11

B07_Stoborough/4312, 12 11 11 -9 12 11 11 -9 11 -9 11 11 11 11 11 11 11 11 11 11 11 11 11 22 11 12 11 11 11 11 11

B07_north_of_Purbeck_hills/2008_002, 11 -9 11 11 11 11 11 11 11 11 11 11 11 11 22 11 -9 12 11 11 11 11 11 12 11 11 11 11 -9 11 11

B07_north_of_Purbeck_hills/2008_003, 11 22 11 11 12 11 11 -9 11 11 11 11 -9 11 12 11 11 11 11 11 -9 11 11 -9 11 11 11 11 11 11 -9

B07_north_of_Purbeck_hills/2008_022, 11 11 -9 11 11 11 -9 12 11 11 11 12 11 11 11 11 12 11 11 11 11 11 11 22 11 11 11 11 11 11 12

B07_north_of_Purbeck_hills/2008_032, 11 11 11 11 12 -9 11 11 11 11 11 11 11 11 22 11 -9 11 11 11 11 11 11 22 11 12 11 11 11 11 11

B07_north_of_Purbeck_hills/2008_039, 11 -9 11 11 12 11 11 11 11 11 11 12 22 11 11 11 12 22 11 11 -9 11 11 11 11 12 11 11 11 11 11

B07_north_of_Purbeck_hills/2008_040, 11 11 11 11 -9 11 12 -9 11 11 11 11 -9 11 12 11 12 11 11 11 11 11 11 -9 11 11 11 11 11 11 -9

B07_north_of_Purbeck_hills/2008_041, 11 11 12 -9 -9 11 -9 11 11 11 11 11 11 11 11 11 12 11 11 11 11 11 11 -9 11 11 12 11 11 11 11

B07_north_of_Purbeck_hills/2008_053, 11 11 11 12 11 11 11 11 11 11 11 11 11 11 11 11 11 11 12 11 -9 11 11 22 -9 11 11 11 11 11 11

B07_north_of_Purbeck_hills/2008_054, 11 11 11 11 11 11 11 11 11 -9 11 11 12 11 11 11 11 11 11 11 -9 11 11 -9 22 11 12 11 -9 11 11

B07_north_of_Purbeck_hills/2008_080, 11 11 11 12 -9 11 11 11 11 11 11 11 12 11 11 11 11 11 11 11 11 11 11 22 -9 11 11 11 11 11 11

B07_north_of_Purbeck_hills/2008_082, 11 11 11 12 12 11 -9 11 11 11 11 11 11 11 11 11 11 11 12 11 11 11 11 12 12 11 11 11 11 11 11

B07_north_of_Purbeck_hills/2008_088, 11 11 11 11 12 11 12 11 11 11 11 11 11 11 11 11 11 11 11 11 11 11 11 22 11 11 11 11 11 11 11

B07_north_of_Purbeck_hills/2008_089, 11 11 11 11 11 11 11 22 11 11 11 11 11 11 11 11 12 11 11 11 -9 11 11 12 11 12 11 11 11 11 12

B07_north_of_Purbeck_hills/2008_108, 11 11 12 11 12 11 12 11 11 11 11 11 11 11 11 11 12 11 11 11 11 11 11 22 11 12 11 11 11 11 11

B07_north_of_Purbeck_hills/2008_121, 11 11 11 11 11 11 11 11 11 -9 11 12 11 11 -9 11 11 11 11 11 11 11 11 11 12 22 11 11 11 11 11

B07_north_of_Purbeck_hills/2008_145, 11 12 11 22 11 11 11 11 11 11 11 12 11 11 11 12 11 11 11 11 11 11 11 22 11 11 11 11 11 11 11

B07_north_of_Purbeck_hills/2008_152, 11 11 11 11 11 11 11 11 11 11 11 11 11 11 12 11 11 11 11 11 11 11 11 11 11 11 11 11 11 11 11

B07_north_of_Purbeck_hills/2008_153, 11 -9 11 11 11 11 22 11 11 11 11 -9 12 11 22 11 -9 11 11 -9 -9 11 11 -9 11 11 22 -9 11 11 11

B07_north_of_Purbeck_hills/2008_156, 12 11 11 11 11 11 12 11 11 11 11 11 11 11 12 12 11 11 11 11 11 11 11 22 11 11 12 11 11 11 11

B07_north_of_Purbeck_hills/2008_162, 11 11 11 12 11 11 -9 11 11 11 11 11 12 11 11 11 11 11 11 11 11 11 11 12 11 11 11 11 11 11 11

B07_north_of_Purbeck_hills/2008_163, 11 11 12 11 11 11 12 11 11 11 11 11 11 -9 -9 11 11 22 11 11 11 11 11 11 11 11 -9 11 11 11 -9

B07_north_of_Purbeck_hills/2008_165, 11 11 12 11 12 11 11 11 12 11 11 12 11 11 12 11 12 11 12 11 11 11 11 12 12 11 11 11 11 11 12

B07_north_of_Purbeck_hills/2008_168, 11 11 11 11 11 11 11 11 11 11 11 11 11 11 11 11 11 12 11 11 11 11 11 12 11 11 22 11 11 11 12

B07_north_of_Purbeck_hills/2008_198, 11 -9 11 -9 12 11 -9 11 11 11 11 11 11 11 22 11 11 11 11 11 11 -9 11 -9 -9 12 11 11 11 11 11

B07_north_of_Purbeck_hills/2008_215, 11 11 11 11 11 12 11 -9 11 11 11 12 12 11 11 11 12 11 11 11 11 11 11 12 11 11 11 11 11 11 12

B07_north_of_Purbeck_hills/2008_220, 11 11 11 11 11 11 -9 11 11 11 11 11 11 11 11 11 11 11 11 11 -9 11 11 -9 -9 11 11 -9 11 11 11

B07_north_of_Purbeck_hills/2008_233, 11 12 12 11 22 11 11 11 11 22 11 12 12 11 11 12 11 11 11 11 11 11 11 11 12 12 11 11 11 11 11

B07_north_of_Purbeck_hills/2008_244, 11 11 11 11 11 11 22 11 11 11 11 12 12 12 11 11 22 22 11 11 11 11 11 12 12 11 11 11 11 11 12

B07_north_of_Purbeck_hills/2008_245, 11 -9 11 -9 11 11 11 11 11 11 11 11 11 11 11 11 12 11 11 11 -9 11 11 -9 -9 11 11 11 11 11 11

B07_north_of_Purbeck_hills/2008_250, -9 11 -9 22 11 11 22 11 11 11 11 11 11 11 -9 11 11 11 -9 11 -9 11 11 -9 11 11 11 -9 11 11 11

B07_north_of_Purbeck_hills/2008_260, 11 11 11 11 11 11 12 11 11 11 11 11 11 12 11 12 11 -9 12 -9 11 11 11 12 12 11 11 11 11 11 11

B07_north_of_Purbeck_hills/2008_261, 11 12 11 11 12 11 11 11 11 11 11 11 12 11 12 11 11 11 12 11 11 11 11 22 11 11 11 11 11 11 11

B07_north_of_Purbeck_hills/2008_264, 11 11 11 12 12 11 11 11 11 11 11 11 11 11 11 11 11 11 12 11 11 11 11 12 11 12 11 11 11 11 11

B07_north_of_Purbeck_hills/2008_269, -9 11 11 11 11 11 11 11 11 11 11 11 -9 11 11 11 12 11 -9 11 -9 11 11 -9 11 -9 11 -9 11 11 12

B07_north_of_Purbeck_hills/2008_272, 11 11 11 11 11 11 11 11 11 12 11 11 11 11 12 11 11 11 11 11 11 12 11 11 11 11 11 11 11 11 11

B07_north_of_Purbeck_hills/2008_283, 11 11 11 12 -9 11 -9 11 11 11 11 11 11 11 -9 11 -9 11 11 11 -9 11 11 -9 12 11 11 11 11 11 11

B07_north_of_Purbeck_hills/2008_309, 11 11 11 11 11 11 11 11 11 11 11 12 12 11 11 11 12 11 12 11 11 12 11 12 11 -9 11 11 11 11 22

B07_north_of_Purbeck_hills/2008_310, 11 12 11 11 11 11 12 -9 11 11 11 11 11 11 11 11 12 12 11 11 11 11 11 12 11 11 11 -9 11 11 11

B07_north_of_Purbeck_hills/2008_313, 11 11 11 11 11 12 11 11 11 12 11 11 12 11 12 11 11 12 22 11 11 11 11 22 11 11 11 12 11 11 11

B07_north_of_Purbeck_hills/2008_327, 11 11 11 -9 11 12 -9 11 11 -9 11 11 11 -9 11 11 11 -9 22 11 -9 11 11 -9 11 11 11 -9 11 11 11

B07_north_of_Purbeck_hills/2008_380, 12 11 11 12 11 11 22 11 11 11 11 11 12 11 11 11 22 11 11 11 11 11 11 22 11 11 12 11 11 11 11

B07_north_of_Purbeck_hills/2008_381, 11 11 11 11 11 11 11 11 11 11 11 12 11 11 11 11 11 11 12 11 11 11 11 11 11 11 11 11 -9 11 12

B07_north_of_Purbeck_hills/2008_385, 11 11 12 -9 -9 11 11 11 11 11 11 11 11 11 11 11 11 11 12 11 11 12 11 -9 11 11 11 11 11 11 11

B07_north_of_Purbeck_hills/2008_392, 11 11 11 -9 11 11 11 11 12 11 11 11 -9 11 11 11 11 11 22 -9 -9 11 -9 -9 -9 11 -9 11 11 11 12

B07_north_of_Purbeck_hills/2008_395, 11 11 11 11 11 11 12 11 11 12 11 11 11 11 12 11 11 11 11 11 11 11 11 12 -9 11 11 12 11 11 11

B07_north_of_Purbeck_hills/2008_433, 11 11 22 -9 -9 11 11 11 11 11 11 11 12 11 11 11 11 11 11 11 -9 -9 11 -9 11 11 -9 11 11 11 22

B07_north_of_Purbeck_hills/2008_434, 11 -9 11 22 22 11 12 11 11 12 11 12 11 11 12 11 11 11 11 11 11 11 11 22 11 12 11 11 11 11 12

B07_north_of_Purbeck_hills/2008_437, 12 12 11 12 11 11 12 11 11 11 12 11 11 11 11 11 11 11 11 11 11 11 11 12 11 12 11 11 11 11 11

B07_north_of_Purbeck_hills/2008_438, 11 11 11 12 12 11 12 11 11 11 11 12 11 11 11 11 11 11 11 11 -9 12 11 22 11 11 12 11 -9 11 12

B07_north_of_Purbeck_hills/2008_439, 11 11 11 11 11 11 11 12 11 11 11 11 11 11 11 12 12 11 11 11 11 11 11 11 11 11 11 11 11 11 22

B07_north_of_Purbeck_hills/2008_440, 11 11 11 12 11 11 -9 11 11 11 11 -9 11 11 11 12 11 11 11 11 11 11 11 -9 12 11 11 11 11 11 11

B07_north_of_Purbeck_hills/2008_441, 11 11 11 12 11 11 11 12 11 11 11 -9 11 11 11 11 -9 11 11 11 11 11 11 -9 11 11 11 11 11 11 11

B07_north_of_Purbeck_hills/2008_445, 12 11 11 11 12 11 11 11 11 11 11 -9 -9 11 11 11 11 22 11 11 11 11 11 22 -9 -9 11 11 -9 11 11

B07_north_of_Purbeck_hills/2008_458, 11 11 11 11 11 11 11 12 11 11 11 11 11 11 12 11 12 11 12 11 11 12 11 -9 11 11 11 11 11 11 12

B07_north_of_Purbeck_hills/2008_471, 11 11 12 11 12 11 11 11 11 11 11 -9 12 11 12 11 -9 11 11 11 11 11 11 12 11 11 11 11 11 11 12

B07_north_of_Purbeck_hills/2008_479, 11 11 11 12 -9 11 11 12 11 11 11 11 11 11 12 11 -9 11 22 11 11 11 11 12 12 11 11 11 11 11 11

B07_north_of_Purbeck_hills/2008_480, 11 11 -9 22 22 -9 -9 11 11 -9 11 -9 -9 11 11 11 11 -9 11 11 11 11 11 -9 -9 11 11 11 11 11 11

B07_north_of_Purbeck_hills/2008_491, 11 11 12 11 11 11 12 12 11 12 11 11 11 11 11 11 11 11 11 11 11 -9 11 22 12 11 11 11 11 11 12

B07_north_of_Purbeck_hills/2008_492, 12 11 11 12 12 11 12 12 11 11 11 -9 11 11 11 11 11 11 11 11 -9 11 11 -9 11 11 11 11 11 11 -9

B07_north_of_Purbeck_hills/2008_495, 11 11 11 11 -9 11 11 11 11 -9 11 11 11 -9 22 11 11 11 11 -9 11 11 11 22 -9 11 11 -9 11 11 11

B07_north_of_Purbeck_hills/2008_497, 11 11 11 22 12 11 11 12 11 11 11 12 11 11 12 11 -9 12 11 11 11 12 11 12 -9 11 11 11 11 11 -9

B07_north_of_Purbeck_hills/2008_499, 11 11 12 11 -9 11 11 11 11 11 11 12 12 11 12 11 11 11 11 11 -9 11 11 11 11 11 11 11 11 11 22

B07_north_of_Purbeck_hills/2008_502, 11 11 11 11 11 11 11 11 11 12 11 11 11 11 11 11 12 11 11 11 11 11 11 12 11 11 11 12 11 11 11

B07_north_of_Purbeck_hills/2008_503, 11 11 12 11 22 11 11 11 11 11 11 11 11 11 11 11 11 11 11 11 11 11 11 -9 12 11 11 11 11 11 11

B07_north_of_Purbeck_hills/2008_522, 11 -9 -9 -9 -9 11 22 11 11 11 22 11 11 11 11 12 11 11 11 11 -9 11 11 -9 11 22 11 11 11 11 11

B07_north_of_Purbeck_hills/2008_525, 11 11 11 12 11 11 11 11 11 12 11 11 11 11 11 11 11 11 11 11 -9 11 11 12 11 11 11 11 11 11 11

B07_north_of_Purbeck_hills/2008_529, 11 11 11 12 11 11 22 11 12 11 11 11 11 11 11 11 11 11 11 11 11 11 11 12 11 11 11 11 11 11 11

B07_north_of_Purbeck_hills/2008_530, 11 11 11 12 12 11 12 12 11 12 11 11 11 11 11 12 12 11 11 11 11 12 11 12 12 11 11 11 11 11 11

B07_north_of_Purbeck_hills/2008_531, 11 22 11 11 11 11 11 11 11 12 11 11 11 11 11 11 11 11 12 11 11 11 11 12 12 11 11 11 11 11 11

B07_north_of_Purbeck_hills/2008_532, 11 11 11 11 11 11 12 11 11 11 11 12 11 11 11 11 11 11 -9 11 -9 11 11 12 11 11 11 11 11 11 12

B07_north_of_Purbeck_hills/2008_537, 11 11 11 11 12 12 11 11 11 11 12 11 11 11 12 11 12 11 11 11 11 11 11 22 11 12 11 11 11 11 12

B07_north_of_Purbeck_hills/2008_538, 11 12 12 11 12 22 12 11 11 12 11 12 11 11 11 11 11 11 11 11 11 11 11 12 11 11 11 11 -9 11 11

B07_north_of_Purbeck_hills/2008_539, 11 11 12 12 12 12 12 11 11 11 11 11 11 11 12 12 11 12 11 11 11 11 11 22 11 12 11 11 11 11 11

B07_north_of_Purbeck_hills/2008_540, 11 12 11 11 11 11 11 11 11 11 11 11 12 11 12 11 11 11 11 11 11 11 11 -9 11 22 11 12 11 11 11

B07_north_of_Purbeck_hills/2008_544, 11 11 11 -9 22 11 -9 11 11 -9 11 11 11 11 12 11 11 11 11 -9 -9 11 11 -9 11 11 11 -9 11 11 -9

B07_north_of_Purbeck_hills/2008_546, 11 12 11 11 11 11 22 11 11 11 11 11 11 11 11 11 12 11 11 11 11 11 11 -9 12 11 11 11 11 11 11

B07_north_of_Purbeck_hills/2008_554, 11 -9 11 -9 11 11 -9 11 11 11 11 -9 22 11 12 11 11 11 11 11 -9 11 11 -9 -9 11 12 11 11 11 22

B07_north_of_Purbeck_hills/2008_575, 11 12 11 11 12 11 22 11 11 22 11 11 -9 -9 11 11 11 -9 11 11 -9 11 11 -9 11 11 11 -9 11 11 11

B07_north_of_Purbeck_hills/2008_576, 11 -9 11 -9 12 11 12 11 11 12 11 12 -9 11 11 -9 12 11 11 11 -9 11 11 22 12 11 11 11 11 11 11

B07_north_of_Purbeck_hills/2008_581, 12 11 11 12 11 11 11 11 11 -9 11 11 11 11 12 11 12 11 11 11 11 11 11 22 11 11 11 11 11 11 11

B07_north_of_Purbeck_hills/2009_003, 11 11 11 11 11 11 12 11 11 11 11 11 11 11 -9 11 11 11 11 -9 11 12 11 12 11 11 11 11 11 11 12

B07_north_of_Purbeck_hills/2009_007, 11 11 11 11 12 11 11 11 11 11 11 11 11 -9 -9 11 12 11 11 11 -9 11 11 12 12 11 11 11 11 -9 11

B07_north_of_Purbeck_hills/2009_009, 11 11 12 11 11 11 12 11 11 12 11 11 12 11 11 11 12 11 11 -9 11 11 11 12 12 11 12 11 11 11 11

B07_north_of_Purbeck_hills/2009_010, 11 -9 11 11 12 11 11 11 11 11 11 11 12 11 11 11 12 11 12 -9 11 11 11 22 11 11 11 11 11 11 11

B07_north_of_Purbeck_hills/2009_011, 12 -9 11 12 11 11 22 12 11 11 11 11 22 11 11 11 12 11 11 11 11 11 11 -9 11 -9 11 11 11 11 11

B07_north_of_Purbeck_hills/2009_012, 11 11 11 22 11 11 12 12 11 11 11 11 11 11 12 11 11 11 11 11 11 12 11 12 12 11 11 11 11 11 11

B07_north_of_Purbeck_hills/2009_015, 11 11 12 11 11 11 11 11 11 -9 11 11 11 11 11 11 11 11 11 11 11 11 11 11 12 11 11 11 11 11 11

B07_north_of_Purbeck_hills/2009_016, 11 11 12 11 -9 11 -9 11 11 11 11 11 22 11 22 11 11 11 11 -9 11 11 11 -9 11 11 11 11 11 11 -9

B07_north_of_Purbeck_hills/2009_017, 11 11 11 11 11 11 11 11 11 11 11 11 11 11 11 11 11 12 11 11 -9 12 11 11 11 11 11 11 11 11 12

B07_north_of_Purbeck_hills/2009_018, 11 12 22 11 11 11 11 11 11 11 11 11 11 11 22 11 11 11 11 11 11 11 11 12 11 11 11 11 11 11 12

B07_north_of_Purbeck_hills/2009_020, 11 11 11 11 11 11 12 11 11 11 11 11 12 11 12 11 11 11 11 11 11 11 11 22 11 11 11 12 11 11 11

B07_north_of_Purbeck_hills/2009_021, 11 11 12 22 11 11 11 11 11 12 11 11 11 11 12 11 12 11 11 11 -9 11 11 12 12 11 11 11 11 11 11

B07_north_of_Purbeck_hills/2009_022, 11 11 11 11 11 11 12 11 11 11 11 11 11 11 11 11 11 12 11 11 11 11 11 12 12 12 11 11 11 11 22

B07_north_of_Purbeck_hills/2009_023, 11 11 11 11 11 12 22 11 11 12 11 11 11 11 11 11 11 11 11 11 11 12 11 22 12 11 11 11 11 11 11

B07_north_of_Purbeck_hills/2009_028, 11 11 11 11 22 11 12 11 11 11 11 11 11 11 11 11 11 12 11 11 11 -9 11 12 11 11 12 11 11 11 11

B07_north_of_Purbeck_hills/2009_029, 11 11 11 12 22 11 11 11 11 12 11 11 12 11 11 11 11 11 11 11 11 11 11 12 11 11 11 12 11 11 12

B07_north_of_Purbeck_hills/2009_038, 12 11 11 12 11 11 11 11 11 11 11 11 11 11 11 11 22 11 11 11 -9 11 11 22 11 11 12 -9 11 11 11

B07_north_of_Purbeck_hills/2009_040, 11 11 12 11 12 11 11 11 11 12 11 11 11 11 11 12 11 22 11 11 11 11 11 22 22 11 11 11 11 11 11

B07_north_of_Purbeck_hills/2009_053, 12 11 11 11 -9 11 11 11 11 11 11 11 12 11 11 11 12 12 11 11 -9 11 11 12 12 11 11 11 11 11 11

B07_north_of_Purbeck_hills/2009_056, 11 11 11 11 12 11 -9 11 11 11 11 11 11 11 11 11 12 11 11 11 11 12 11 11 11 12 11 12 11 11 11

B07_north_of_Purbeck_hills/2009_057, 12 11 11 11 11 11 22 11 11 12 11 11 11 11 11 11 11 12 11 11 11 11 11 22 12 11 11 11 11 11 11

B07_north_of_Purbeck_hills/2009_058, 11 11 11 12 11 11 11 11 11 11 11 22 11 11 12 11 11 11 11 11 11 11 11 12 11 11 11 11 11 11 12

B07_north_of_Purbeck_hills/2009_061, 12 11 12 12 11 11 12 11 11 11 11 11 11 11 11 12 11 11 11 11 -9 11 11 12 12 11 11 11 11 11 11

B07_north_of_Purbeck_hills/2009_062, 11 12 11 11 11 11 11 11 11 11 11 11 12 11 11 11 11 11 12 11 11 11 11 12 11 11 11 11 11 11 11

B07_north_of_Purbeck_hills/2009_063, 12 11 11 11 11 11 11 11 11 11 11 11 22 11 11 11 12 11 11 11 11 12 11 22 11 11 11 11 11 11 11

B07_north_of_Purbeck_hills/2009_067, 11 11 11 11 11 11 11 11 11 11 11 11 11 11 12 11 12 11 11 11 11 11 11 11 12 -9 -9 11 -9 11 11

B07_north_of_Purbeck_hills/2009_070, 11 11 11 11 11 11 -9 11 11 12 11 11 11 11 11 11 11 11 11 11 11 11 11 12 11 11 11 11 11 11 11

B07_north_of_Purbeck_hills/2009_071, 11 11 11 12 11 11 12 11 11 11 11 12 12 11 11 11 11 11 11 11 11 11 11 22 11 11 11 11 11 11 12

B07_north_of_Purbeck_hills/2009_072, 11 11 11 11 11 11 12 11 11 11 11 11 12 11 11 11 12 12 11 11 11 11 11 12 11 11 11 11 11 11 11

B07_north_of_Purbeck_hills/2009_073, -9 12 11 12 12 11 12 11 11 11 11 11 11 11 11 12 11 11 11 11 11 11 11 22 11 11 11 -9 11 11 11

B07_north_of_Purbeck_hills/2009_074, 11 11 11 11 11 12 12 11 11 11 11 12 11 11 12 11 12 11 11 11 11 12 11 12 11 11 12 11 11 22 12

B07_north_of_Purbeck_hills/2009_076, 11 11 12 11 11 11 11 11 11 11 11 11 11 11 12 11 11 11 11 11 11 11 11 -9 11 11 11 11 11 11 11

B07_north_of_Purbeck_hills/2009_077, 11 11 11 11 11 11 11 11 11 11 11 11 11 11 11 11 11 11 11 11 11 11 11 11 -9 12 11 11 11 11 11

B07_north_of_Purbeck_hills/2009_079, 11 11 11 12 22 11 11 11 11 11 11 11 11 11 11 11 12 11 11 11 11 11 11 22 11 11 12 11 11 11 11

B07_north_of_Purbeck_hills/2009_082, 11 11 11 11 12 11 11 12 11 12 11 11 11 11 11 11 12 11 11 11 11 11 11 22 12 11 11 11 11 11 11

B07_north_of_Purbeck_hills/2009_085, 11 12 11 11 11 11 11 11 12 11 11 11 11 11 12 11 11 11 12 11 11 12 11 12 11 11 11 11 11 11 22

B07_north_of_Purbeck_hills/2009_086, 12 11 11 11 11 11 12 11 11 11 11 11 11 11 11 11 11 11 11 11 11 11 11 22 11 11 11 12 11 11 12

B07_north_of_Purbeck_hills/2009_090, 11 11 12 11 11 11 12 11 11 11 11 12 11 11 11 11 11 11 11 11 11 11 11 12 11 11 11 11 11 11 22

B07_north_of_Purbeck_hills/2009_092, 11 11 12 11 11 12 11 11 11 11 11 12 11 11 11 11 12 11 11 11 11 11 11 22 12 11 11 11 11 11 11

B07_north_of_Purbeck_hills/2009_093, 11 -9 11 12 12 11 -9 11 12 11 11 22 -9 11 11 11 11 11 -9 11 -9 12 11 22 -9 12 11 11 11 11 -9

B07_north_of_Purbeck_hills/2009_094, 11 11 11 11 11 11 12 11 11 11 11 12 11 11 11 11 11 11 12 11 11 11 11 12 11 11 11 12 11 11 12

B07_north_of_Purbeck_hills/2009_096, 12 11 11 11 11 12 11 12 11 11 11 11 12 12 11 11 -9 11 11 11 11 11 11 22 11 11 11 11 11 11 11

B07_north_of_Purbeck_hills/2009_097, 11 11 12 12 12 11 11 11 11 11 11 11 11 11 22 12 11 11 11 11 11 12 11 12 11 11 12 11 -9 11 11

B07_north_of_Purbeck_hills/2009_100, 12 11 11 11 22 12 12 11 11 11 11 11 11 11 11 11 12 12 11 11 -9 11 11 12 11 11 12 11 11 11 11

B07_north_of_Purbeck_hills/2009_101, 11 11 11 12 11 11 12 11 11 11 11 11 12 11 11 12 11 12 11 11 -9 11 11 12 11 11 11 11 11 11 11

B07_north_of_Purbeck_hills/2009_104, 11 11 11 11 11 11 12 11 11 11 11 22 11 11 11 11 12 11 11 11 11 11 11 22 11 12 11 11 11 11 22

B07_north_of_Purbeck_hills/2009_105, 11 11 12 11 11 11 12 11 11 12 11 11 12 11 12 11 11 12 11 11 11 11 11 22 11 11 11 11 11 11 12

B07_north_of_Purbeck_hills/2009_107, 11 11 12 12 11 11 11 11 11 11 11 11 12 11 11 12 11 11 11 11 11 11 11 12 11 11 11 11 11 11 12

B07_north_of_Purbeck_hills/2009_109, 11 11 11 11 11 11 11 11 11 11 11 11 11 11 11 11 11 11 11 11 11 11 11 12 22 12 11 11 11 11 12

B07_north_of_Purbeck_hills/2009_116, 11 11 11 12 12 11 11 11 12 11 11 22 11 11 11 11 11 11 12 11 11 11 11 22 12 12 11 11 11 11 22

B07_north_of_Purbeck_hills/2009_122, 11 11 11 11 12 11 12 11 11 11 11 11 11 11 11 11 11 11 11 11 11 12 11 22 11 11 11 12 11 11 12

B07_north_of_Purbeck_hills/2009_123, 11 11 11 11 -9 -9 11 11 11 11 11 11 12 -9 11 12 11 11 11 11 -9 11 11 -9 12 11 11 11 11 11 11

B07_north_of_Purbeck_hills/2009_127, 11 11 11 11 11 11 11 11 11 12 11 11 11 11 11 12 11 12 12 11 11 11 11 11 11 11 11 11 11 11 11

B07_north_of_Purbeck_hills/2009_128, 11 12 11 11 11 11 11 12 11 11 12 12 11 11 11 11 12 11 11 11 11 11 11 12 11 11 11 11 11 11 22

B07_north_of_Purbeck_hills/2009_131, 11 11 11 11 12 11 11 11 11 11 11 22 12 11 11 11 11 11 11 11 11 11 11 12 11 12 11 11 11 11 11

B07_north_of_Purbeck_hills/2009_132, 11 12 12 11 12 11 22 11 11 11 11 11 11 11 12 11 11 11 12 11 11 12 11 22 11 12 11 12 11 11 12

B07_north_of_Purbeck_hills/2009_133, 11 11 11 11 -9 11 11 11 11 11 11 11 11 11 11 11 11 12 11 11 11 12 11 22 11 11 11 11 11 22 11

B07_north_of_Purbeck_hills/2009_134, 11 11 12 12 12 11 11 11 11 11 11 11 11 11 12 11 12 11 11 11 11 11 11 12 11 12 11 11 11 11 11

B07_north_of_Purbeck_hills/2009_135, 12 11 11 11 11 11 11 11 -9 11 11 22 12 11 11 11 -9 -9 11 11 -9 -9 11 -9 -9 11 12 11 11 11 22

B07_north_of_Purbeck_hills/2009_136, 12 12 11 11 12 12 11 11 11 22 11 11 11 11 12 11 11 11 12 11 11 11 11 22 11 11 11 11 11 11 11

B07_north_of_Purbeck_hills/2009_137, 11 11 11 11 11 11 -9 11 11 12 11 11 11 11 22 11 11 -9 12 -9 -9 11 11 22 11 11 11 11 11 11 11

B07_north_of_Purbeck_hills/2009_140, 11 12 11 12 12 11 12 11 12 11 11 11 12 11 11 11 11 11 11 11 11 11 11 12 11 11 11 11 11 11 12

B07_north_of_Purbeck_hills/2009_141, 11 11 11 11 11 11 11 11 11 11 11 11 11 11 12 11 11 11 11 11 11 11 11 12 11 11 11 12 11 11 11

B07_north_of_Purbeck_hills/2009_142, 11 11 11 11 11 11 11 11 11 11 11 11 11 11 11 11 22 11 11 11 11 11 11 11 12 11 11 11 11 11 11

B07_north_of_Purbeck_hills/2009_144, 12 11 11 11 11 11 11 12 11 11 11 11 11 11 12 11 11 11 11 11 11 11 11 12 11 11 11 11 11 11 11

B07_north_of_Purbeck_hills/2009_145, 11 11 12 12 11 11 12 11 11 11 11 11 12 11 12 11 11 11 11 11 11 11 11 12 12 11 11 11 11 11 11

B07_north_of_Purbeck_hills/2009_147, 11 11 11 11 11 11 11 12 11 11 11 11 12 11 12 11 11 11 12 11 -9 11 11 -9 11 11 11 11 11 11 11

B07_north_of_Purbeck_hills/2009_149, 11 11 11 11 12 11 11 11 11 12 11 11 11 11 12 11 11 11 11 11 11 11 11 22 11 12 11 11 11 11 12

B07_north_of_Purbeck_hills/2009_150, 11 -9 12 11 11 11 22 22 11 11 11 12 11 11 12 11 12 11 11 11 11 11 11 22 11 11 11 11 -9 11 11

B07_north_of_Purbeck_hills/2009_154, 11 -9 12 11 12 11 11 11 11 11 11 11 11 11 22 11 11 11 11 11 -9 11 11 -9 11 11 11 11 11 11 11

B07_north_of_Purbeck_hills/2009_157, 11 11 11 11 11 11 12 12 11 11 11 11 11 11 11 11 12 11 11 11 11 11 11 12 11 11 11 11 11 11 22

B07_north_of_Purbeck_hills/2009_162, 11 11 11 11 11 11 12 12 11 11 11 11 11 11 11 11 11 12 12 11 -9 11 11 11 11 11 11 11 11 11 11

B07_north_of_Purbeck_hills/2009_163, 11 11 11 11 12 11 22 11 11 11 11 11 11 11 11 11 12 11 11 11 11 11 11 22 22 11 11 11 11 11 12

B07_north_of_Purbeck_hills/2009_164, 11 11 11 12 11 11 11 11 11 12 12 11 12 11 11 11 11 11 11 11 11 11 11 22 11 11 11 11 11 11 11

B07_north_of_Purbeck_hills/2009_166, 11 11 12 11 11 11 12 11 11 11 11 11 11 11 11 12 12 11 12 11 11 11 11 12 11 11 11 12 11 11 11

B07_north_of_Purbeck_hills/2009_170, 11 12 11 11 11 11 11 11 11 11 11 11 12 12 12 11 12 11 11 11 11 12 11 12 11 11 11 11 11 11 11

B07_north_of_Purbeck_hills/2009_171, 12 -9 12 12 11 11 11 11 11 11 11 11 11 11 11 11 12 11 11 11 11 11 11 11 11 11 11 11 11 11 11

B07_north_of_Purbeck_hills/2009_172, 11 11 11 11 12 11 12 11 11 11 11 11 11 11 11 11 11 11 11 11 11 11 11 22 11 11 11 11 11 11 11

B07_north_of_Purbeck_hills/2009_176, 11 11 11 11 11 11 12 11 11 11 11 11 11 11 11 11 11 11 11 11 11 11 11 12 11 11 11 11 11 11 12

B07_north_of_Purbeck_hills/2009_177, 12 12 11 12 11 11 12 11 11 11 11 11 11 11 12 11 11 11 11 11 11 11 11 12 12 12 11 11 11 11 11

B07_north_of_Purbeck_hills/2009_179, 11 11 11 11 12 11 11 11 12 11 11 11 12 11 11 11 12 12 11 11 11 11 11 12 11 11 11 11 11 11 12

B07_north_of_Purbeck_hills/2009_190, 11 -9 12 11 12 -9 12 11 11 -9 11 12 11 11 11 11 11 11 11 11 -9 11 11 22 11 11 11 11 11 11 11

B07_north_of_Purbeck_hills/2009_191, 11 11 11 12 12 11 12 11 11 11 11 12 12 11 11 12 11 11 11 11 11 11 11 12 11 11 11 11 -9 11 12

B07_north_of_Purbeck_hills/2009_192, 11 11 11 11 12 11 12 12 12 11 11 22 11 11 11 11 -9 11 11 11 -9 11 11 22 22 11 11 11 11 11 12

B07_north_of_Purbeck_hills/2009_194, 11 11 12 11 22 11 12 12 11 12 11 12 22 11 11 11 11 11 11 11 11 11 11 22 12 12 11 11 11 11 11

B07_north_of_Purbeck_hills/2009_195, 11 11 11 11 12 11 12 11 12 11 11 11 12 11 11 11 11 11 11 11 -9 11 11 22 22 11 12 11 11 11 12

B07_north_of_Purbeck_hills/2009_199, 11 11 11 11 11 11 11 12 11 22 11 -9 11 11 11 11 -9 -9 11 11 -9 11 11 -9 11 12 11 11 11 11 11

B07_north_of_Purbeck_hills/2009_207, 11 11 11 22 22 12 12 11 11 11 11 11 11 11 11 11 11 11 11 11 11 11 11 11 12 12 11 11 11 11 11

B07_north_of_Purbeck_hills/2009_218, 11 11 11 11 11 11 12 12 11 11 11 11 11 11 11 12 12 11 12 11 11 12 11 22 12 11 11 11 11 11 22

B07_north_of_Purbeck_hills/2009_219, 11 11 11 11 11 11 12 11 11 11 11 11 11 12 12 11 11 12 22 11 11 11 11 12 12 11 11 12 11 11 11

B07_north_of_Purbeck_hills/2009_223, 11 11 11 12 12 11 11 11 11 11 11 12 11 11 11 11 11 11 11 11 11 12 11 12 11 12 11 11 11 11 12

B07_north_of_Purbeck_hills/2009_224, 11 11 11 12 11 11 12 11 11 11 11 11 11 11 11 11 12 11 11 11 11 11 11 12 11 11 11 11 11 11 11

B07_north_of_Purbeck_hills/2009_225, 11 11 11 11 22 12 11 11 11 -9 11 11 12 11 11 11 11 11 11 11 -9 11 11 22 12 12 11 11 11 11 11

B07_north_of_Purbeck_hills/2009_227, 11 11 12 11 11 11 11 11 11 11 11 12 11 11 12 12 11 11 11 11 -9 12 11 11 11 11 11 11 11 11 11

B07_north_of_Purbeck_hills/2009_229, 11 11 11 12 12 11 11 11 11 11 11 11 11 11 12 11 12 11 12 11 11 22 11 22 12 11 11 12 11 11 11

B07_north_of_Purbeck_hills/2009_231, -9 11 11 11 11 11 11 11 11 11 11 11 11 11 11 11 11 -9 11 11 11 11 11 12 12 -9 11 11 11 11 11

B07_north_of_Purbeck_hills/2009_232, 11 11 11 11 22 11 22 11 11 12 12 12 11 11 11 11 12 11 11 -9 11 11 11 -9 11 11 11 11 11 11 11

B07_north_of_Purbeck_hills/2009_233, 11 11 11 11 11 11 11 11 11 12 11 11 -9 11 11 11 11 11 11 11 11 12 11 -9 11 12 11 11 11 -9 12

B07_north_of_Purbeck_hills/2009_234, 11 11 11 11 12 11 11 11 11 11 11 22 -9 11 11 11 12 -9 11 11 11 11 11 12 11 11 11 11 11 -9 12

B07_north_of_Purbeck_hills/2009_238, 11 11 11 11 12 11 11 11 11 12 11 11 11 11 12 11 11 11 11 11 11 12 11 12 12 11 11 12 11 -9 11

B07_north_of_Purbeck_hills/2009_239, 11 11 12 11 12 11 12 11 11 11 11 11 11 11 11 11 11 11 12 11 11 12 11 12 11 11 12 11 11 11 22

B07_north_of_Purbeck_hills/2009_242, -9 11 11 11 22 11 11 11 11 12 11 11 12 11 11 11 11 12 11 11 11 11 11 -9 11 12 11 11 11 11 11

B07_north_of_Purbeck_hills/2009_244, 11 11 11 11 11 11 11 11 11 11 11 11 11 12 11 11 11 11 11 11 11 11 11 12 12 12 11 11 11 11 11

B07_north_of_Purbeck_hills/2009_246, 11 11 12 12 11 11 11 11 11 11 11 11 12 11 11 11 12 11 11 11 11 12 11 12 11 11 12 12 11 11 22

B07_north_of_Purbeck_hills/2009_248, 11 11 11 12 12 11 22 11 11 11 11 11 11 11 11 11 11 11 11 11 -9 11 11 22 11 11 12 11 11 11 11

B07_north_of_Purbeck_hills/2009_249, 11 11 11 11 -9 11 11 11 11 12 12 11 11 11 -9 11 12 11 11 11 11 12 11 22 11 12 11 11 11 11 11

B07_north_of_Purbeck_hills/2009_250, 11 11 12 12 12 11 11 12 11 11 11 11 11 11 11 11 11 11 11 11 11 11 11 11 11 12 11 11 11 11 11

B07_north_of_Purbeck_hills/2009_251, 11 11 11 11 12 11 22 12 11 12 12 11 11 11 11 11 11 11 11 11 -9 11 11 12 12 11 11 11 11 11 -9

B07_north_of_Purbeck_hills/2009_252, 11 11 11 12 12 12 11 22 11 11 11 12 11 11 11 11 22 22 12 11 11 11 11 22 12 11 11 11 11 11 12

B07_north_of_Purbeck_hills/2009_253, 11 11 11 11 12 11 11 11 11 11 11 12 22 11 11 11 11 11 11 11 -9 11 11 22 11 11 11 11 11 11 22

B07_north_of_Purbeck_hills/2009_254, 11 -9 11 12 11 11 11 11 11 11 11 11 11 11 12 11 -9 11 12 11 -9 11 11 12 12 11 11 11 11 11 12

B07_north_of_Purbeck_hills/2009_255, 11 11 11 12 11 11 12 12 11 12 11 12 12 11 11 11 11 12 12 11 11 11 11 22 11 11 11 12 11 11 12

B07_north_of_Purbeck_hills/2009_257, 11 11 11 11 12 11 11 12 11 11 11 11 11 11 11 11 12 11 12 11 11 11 11 12 11 11 12 11 11 11 11

B07_north_of_Purbeck_hills/2009_265, 11 11 11 11 11 11 11 11 11 11 11 12 12 11 11 11 11 11 11 11 11 11 11 12 11 11 11 11 11 11 11

B07_north_of_Purbeck_hills/2009_266, 11 11 11 12 12 11 11 11 11 12 11 11 12 11 11 11 11 11 11 11 11 22 11 12 11 12 11 12 11 11 12

B07_north_of_Purbeck_hills/2009_272, 11 11 11 11 12 11 12 11 11 12 11 11 12 11 11 11 11 11 11 11 11 11 11 12 12 11 12 11 11 11 12

B07_north_of_Purbeck_hills/2009_273, 11 11 11 11 11 11 11 11 11 11 11 11 11 12 11 11 11 11 11 11 11 11 11 22 11 11 11 11 11 11 22

B07_north_of_Purbeck_hills/2009_274, 11 11 11 12 11 11 11 11 11 11 11 11 11 11 22 11 11 11 12 11 11 11 11 12 -9 11 11 11 11 11 12

B07_north_of_Purbeck_hills/2009_275, 11 11 11 11 11 11 -9 12 11 11 11 11 11 12 11 11 -9 11 11 11 11 11 11 11 12 11 11 11 11 11 11

B07_north_of_Purbeck_hills/2009_276, 11 11 11 11 11 11 12 11 11 11 11 12 11 11 12 11 12 11 11 11 11 12 11 12 11 11 11 11 11 11 12

B07_north_of_Purbeck_hills/2009_277, 12 12 11 11 12 12 11 11 11 11 11 11 11 12 11 11 11 12 11 11 11 11 11 22 12 11 11 11 11 11 12

B07_north_of_Purbeck_hills/2009_278, 11 11 12 11 11 12 11 11 11 11 11 11 11 11 11 11 12 11 12 11 11 11 11 12 12 11 11 11 11 11 11

B07_north_of_Purbeck_hills/2009_280, 12 11 11 12 11 11 11 11 11 11 11 11 11 11 12 12 11 11 11 11 11 -9 11 12 12 12 11 11 11 11 12

B07_north_of_Purbeck_hills/2009_281, 11 11 11 12 11 12 12 11 11 11 11 11 12 11 11 11 11 11 11 11 -9 11 11 12 12 11 11 11 11 11 11

B07_north_of_Purbeck_hills/2009_282, 12 11 11 22 11 11 11 11 11 22 11 11 11 11 12 11 11 11 11 11 11 11 11 22 11 11 11 11 11 11 11

B07_north_of_Purbeck_hills/2009_283, 11 11 11 11 11 11 11 11 11 11 11 11 11 11 11 11 12 11 12 11 11 12 11 12 11 11 11 11 11 11 11

B07_north_of_Purbeck_hills/2009_286, 11 11 11 11 11 22 11 11 11 -9 11 11 11 11 -9 11 -9 12 11 11 11 11 11 11 22 12 11 12 11 11 22

B07_north_of_Purbeck_hills/2009_287, 11 11 11 12 11 11 11 -9 11 12 12 11 12 11 11 11 11 11 12 11 11 11 11 12 -9 22 12 11 11 11 11

B07_north_of_Purbeck_hills/2009_288, 12 12 11 11 11 11 22 11 11 11 11 11 11 11 12 11 11 11 12 11 11 11 11 12 11 12 12 11 11 11 11

B07_north_of_Purbeck_hills/2009_289, 12 11 11 11 11 11 11 11 11 11 12 12 11 11 11 11 12 11 22 11 11 11 11 22 11 11 11 11 11 11 11

B07_north_of_Purbeck_hills/2009_292, 11 11 11 12 22 11 11 11 11 11 11 11 22 11 11 11 11 11 11 11 11 11 11 12 -9 12 11 11 11 11 11

B07_north_of_Purbeck_hills/2009_293, 11 11 11 11 11 11 11 11 11 11 11 12 12 11 11 12 11 11 11 11 11 11 11 -9 -9 11 11 11 11 11 12

B07_north_of_Purbeck_hills/2009_294, 11 -9 12 11 -9 11 11 11 11 11 11 11 11 11 11 12 12 11 12 11 11 11 11 12 11 11 11 11 11 11 -9

B07_north_of_Purbeck_hills/2009_295, 12 11 22 11 12 11 12 11 11 11 11 11 11 11 11 11 11 11 11 11 11 11 11 12 12 11 12 11 -9 11 12

B07_north_of_Purbeck_hills/2009_296, 11 11 11 11 11 11 11 11 11 11 11 11 11 11 11 11 12 12 22 11 11 11 11 12 12 11 11 11 11 11 11

B07_north_of_Purbeck_hills/2009_299, -9 12 11 22 12 11 11 12 11 11 22 11 12 11 -9 11 12 11 12 11 11 11 11 22 -9 12 -9 -9 11 11 11

B07_north_of_Purbeck_hills/2009_304, 11 11 11 11 11 11 11 12 11 11 11 12 11 11 11 12 11 11 22 11 11 11 11 11 11 11 12 11 11 11 11

B07_north_of_Purbeck_hills/2009_306, 11 11 12 12 11 11 11 12 11 11 11 11 11 11 11 12 11 11 11 11 11 11 11 11 11 12 11 11 11 11 12

B07_north_of_Purbeck_hills/2009_310, 11 11 11 12 11 11 11 11 11 12 11 11 12 11 12 12 11 11 12 11 11 11 11 22 11 11 11 11 11 11 11

B07_north_of_Purbeck_hills/2009_311, 12 11 11 22 11 12 11 11 11 12 11 11 12 11 11 11 11 11 11 11 11 11 11 22 12 11 11 11 11 11 11

B07_north_of_Purbeck_hills/2009_315, 11 11 11 11 11 11 11 11 11 11 11 11 -9 11 11 11 11 11 22 11 -9 -9 11 -9 -9 11 11 11 11 11 11

B07_north_of_Purbeck_hills/2009_316, 11 -9 11 11 -9 11 -9 11 11 11 11 11 11 11 11 11 11 11 11 11 11 11 11 12 11 11 11 11 11 11 11

B07_north_of_Purbeck_hills/2009_317, 11 12 11 12 12 11 11 11 11 12 11 11 11 11 12 11 11 11 11 11 11 11 11 22 12 12 11 11 11 11 12

B07_north_of_Purbeck_hills/2009_318, 11 11 11 11 11 12 11 11 11 11 11 11 12 11 12 11 11 12 12 11 11 12 11 11 11 11 11 11 11 11 11

B07_north_of_Purbeck_hills/2009_319, 11 11 11 11 11 12 11 -9 11 11 11 -9 -9 11 11 11 11 11 11 11 11 -9 11 -9 11 12 11 11 11 11 -9

B07_north_of_Purbeck_hills/2009_321, 11 12 11 11 11 11 12 11 11 11 11 11 11 11 11 11 11 11 12 11 11 11 11 12 12 11 11 11 11 11 12

B07_north_of_Purbeck_hills/2009_322, 11 12 11 11 12 12 11 11 11 12 11 11 11 12 11 11 11 11 11 11 11 11 11 12 -9 11 11 11 11 11 22

B07_north_of_Purbeck_hills/2009_323, 11 11 11 11 11 11 12 11 11 -9 12 11 11 11 11 11 11 12 12 11 11 11 11 12 11 11 11 11 -9 11 11

B07_north_of_Purbeck_hills/2009_325, 12 11 11 11 12 11 11 11 11 11 11 11 11 11 11 11 12 11 12 11 11 11 11 12 22 -9 11 11 11 11 11

B07_north_of_Purbeck_hills/2009_326, 11 11 11 11 12 11 12 11 11 12 11 11 11 11 11 11 -9 12 11 11 11 11 11 22 22 11 11 12 11 11 11

B07_north_of_Purbeck_hills/2009_327, 11 11 11 11 12 11 11 12 11 12 11 11 22 11 22 12 22 11 11 11 11 11 11 12 11 11 11 11 11 11 11

B07_north_of_Purbeck_hills/2009_329, 11 11 12 12 12 11 -9 12 11 11 11 12 12 11 12 12 12 12 11 11 11 11 11 22 11 12 11 11 11 11 12

B07_north_of_Purbeck_hills/2009_330, -9 -9 11 11 11 11 -9 11 11 11 11 11 12 11 11 12 11 11 11 11 11 11 11 -9 11 11 11 11 11 11 11

B07_north_of_Purbeck_hills/2009_331, 11 12 11 12 11 11 12 11 11 12 11 11 11 11 11 11 11 11 11 11 -9 11 11 12 12 11 11 11 11 11 12

B07_north_of_Purbeck_hills/2009_332, 11 22 12 11 22 11 11 11 11 -9 11 11 11 11 11 11 12 11 11 11 11 11 11 22 12 11 11 11 11 11 11

B07_north_of_Purbeck_hills/2009_333, 11 11 11 11 11 11 11 11 11 11 11 12 11 11 11 12 11 11 11 11 11 11 11 -9 -9 11 11 11 11 11 11

B07_north_of_Purbeck_hills/2009_340, 11 11 12 11 11 11 11 11 11 12 11 12 12 11 11 11 11 12 11 11 11 12 11 22 11 12 11 11 11 11 22

B07_north_of_Purbeck_hills/2009_342, 12 11 11 11 11 11 11 11 11 11 11 11 11 11 12 11 11 11 11 11 11 11 11 22 11 11 11 11 11 11 11

B07_north_of_Purbeck_hills/2009_343, 11 11 12 11 11 12 12 11 11 -9 11 11 11 11 11 11 11 11 11 11 11 11 11 12 11 11 11 11 11 11 11

B07_north_of_Purbeck_hills/2009_344, 11 11 11 11 11 11 11 11 11 11 11 11 11 11 11 11 11 12 11 11 11 -9 11 -9 11 12 11 -9 11 11 12

B07_north_of_Purbeck_hills/2009_345, 11 12 11 11 11 11 11 11 11 12 11 11 11 11 11 11 12 11 11 11 11 11 11 -9 11 11 11 12 11 11 11

B07_north_of_Purbeck_hills/2009_346, 12 11 11 11 11 11 12 11 12 11 11 12 11 12 12 11 11 11 12 11 11 11 11 12 -9 11 11 11 11 11 12

B07_north_of_Purbeck_hills/2009_347, 11 11 11 12 11 12 11 11 11 12 11 12 12 11 11 11 12 11 11 11 11 12 11 22 12 11 11 11 11 11 11

B07_north_of_Purbeck_hills/2009_348, 11 11 12 12 12 11 12 11 11 11 11 11 11 11 11 11 11 11 11 11 11 11 11 22 12 11 12 11 11 11 11

B07_north_of_Purbeck_hills/2009_351, 11 12 12 22 11 11 11 11 11 11 11 11 11 11 11 11 11 11 11 11 11 11 11 12 12 12 11 11 11 11 11

B07_north_of_Purbeck_hills/2009_358, 11 -9 11 12 11 11 11 11 -9 -9 11 -9 12 11 22 11 11 11 11 11 -9 12 11 -9 -9 12 11 -9 11 -9 22

B07_north_of_Purbeck_hills/2009_359, 11 12 12 11 -9 11 -9 11 11 12 11 11 12 11 11 -9 12 11 11 11 11 11 11 12 12 11 11 -9 11 -9 -9

B07_north_of_Purbeck_hills/2009_363, 12 11 11 11 11 11 12 11 11 11 11 11 12 11 12 11 11 11 12 11 11 11 11 22 12 11 11 11 11 11 12

B07_north_of_Purbeck_hills/2009_365, 11 11 11 22 12 -9 11 12 12 12 11 11 11 11 11 11 11 11 11 11 11 11 11 11 -9 11 11 11 11 11 12

B07_north_of_Purbeck_hills/2009_368, 11 11 12 -9 12 11 11 12 11 11 11 12 11 11 22 11 11 11 11 11 11 12 11 11 12 11 11 11 11 12 11

B07_north_of_Purbeck_hills/2009_369, 12 11 11 11 12 11 11 12 11 -9 11 11 11 11 12 11 22 11 12 11 11 11 11 12 11 11 11 11 11 11 12

B07_north_of_Purbeck_hills/2009_371, 11 11 11 11 12 11 12 12 11 -9 11 11 12 11 11 11 22 12 11 11 -9 11 11 12 11 11 12 11 11 11 11

B07_north_of_Purbeck_hills/2009_372, 11 -9 11 12 12 11 12 11 11 12 11 22 11 11 22 12 11 11 11 11 11 11 11 12 12 12 11 11 11 11 12

B07_north_of_Purbeck_hills/2009_380, 11 11 11 11 12 11 11 11 11 11 11 11 11 11 11 11 12 11 11 11 11 11 11 22 11 11 11 11 11 11 11

B07_north_of_Purbeck_hills/2009_384, 11 11 11 12 11 11 22 11 11 11 11 11 -9 11 12 11 11 11 11 11 -9 11 11 11 12 11 11 11 11 11 11

B07_north_of_Purbeck_hills/2009_386, 12 11 11 12 11 11 11 11 11 -9 11 12 11 11 -9 11 12 11 12 11 11 11 11 12 11 11 11 11 11 11 11

B07_north_of_Purbeck_hills/2009_387, 11 11 12 12 11 11 12 11 11 11 11 11 11 11 12 11 11 11 11 11 11 12 11 11 11 11 11 11 11 11 11

B07_north_of_Purbeck_hills/2009_388, 11 11 11 11 11 11 22 11 11 11 11 11 12 11 11 11 12 11 11 11 11 11 11 22 11 11 11 11 11 11 11

B07_north_of_Purbeck_hills/2009_389, 12 11 11 12 11 12 11 11 11 11 11 11 12 11 11 11 11 11 12 11 11 11 11 12 11 11 11 11 11 11 12

B07_north_of_Purbeck_hills/2009_390, 12 11 -9 11 11 11 11 11 11 11 11 11 12 11 11 -9 11 12 12 11 11 12 11 12 12 11 11 11 11 11 12

B07_north_of_Purbeck_hills/2009_393, 11 11 11 11 11 11 12 11 11 11 11 11 11 11 12 11 11 11 11 11 11 11 11 -9 11 11 11 11 11 11 12

B07_north_of_Purbeck_hills/2009_394, 11 11 11 11 22 11 11 11 11 11 11 12 11 11 11 11 11 11 11 11 11 11 11 12 12 11 12 11 11 11 12

B07_north_of_Purbeck_hills/2009_395, 11 11 11 11 12 11 12 11 11 11 11 11 -9 11 11 -9 12 11 11 11 11 -9 11 -9 11 22 11 11 -9 11 12

B07_north_of_Purbeck_hills/2009_396, 11 12 11 12 22 22 12 11 11 12 11 11 22 11 11 11 11 11 11 11 11 11 11 12 11 11 11 11 11 11 11

B07_north_of_Purbeck_hills/2009_397, 11 11 11 11 -9 11 22 11 11 11 11 11 11 11 12 11 12 11 11 11 -9 11 11 22 11 11 11 11 11 12 12

B07_north_of_Purbeck_hills/2009_398, 11 11 11 11 12 11 12 11 11 11 11 12 12 11 11 11 11 12 11 11 11 11 11 12 12 11 11 11 11 11 22

B07_north_of_Purbeck_hills/2009_399, 12 11 12 11 11 11 11 11 11 11 11 11 11 12 11 11 11 11 11 11 11 12 11 12 11 11 11 11 11 11 11

B07_north_of_Purbeck_hills/2009_400, 11 11 11 11 11 11 11 12 11 11 11 11 -9 11 11 11 12 11 12 11 -9 11 11 22 11 11 -9 12 11 11 11

B07_north_of_Purbeck_hills/2009_401, 11 11 11 11 12 11 11 12 11 11 12 12 11 11 11 11 11 11 11 11 11 11 11 12 12 11 11 12 11 11 12

B07_north_of_Purbeck_hills/2009_402, 11 11 11 11 11 11 11 11 11 11 11 11 11 11 11 -9 11 11 -9 11 -9 11 11 22 11 12 11 11 -9 11 11

B07_north_of_Purbeck_hills/2009_405, 11 11 11 11 12 12 11 11 11 11 11 11 12 11 11 11 11 11 11 11 11 11 11 12 11 12 11 11 11 11 11

B07_north_of_Purbeck_hills/2009_407, 11 11 11 11 22 12 12 11 11 11 11 11 11 12 11 11 -9 11 11 11 11 11 11 22 11 11 11 11 11 11 11

B07_north_of_Purbeck_hills/2009_408, 11 11 11 11 12 11 12 11 11 11 11 11 22 11 12 12 11 12 11 11 11 11 11 12 12 12 11 11 11 11 11

B07_north_of_Purbeck_hills/2009_410, 11 12 11 11 11 11 12 11 11 11 11 11 12 11 12 12 11 11 11 11 11 22 11 12 11 11 11 11 11 11 11

B07_north_of_Purbeck_hills/2009_411, 11 12 11 11 12 12 11 12 11 12 11 11 11 12 11 11 12 11 11 11 11 11 11 12 11 11 11 11 11 11 11

B07_north_of_Purbeck_hills/2009_417, 11 11 11 12 11 11 12 11 11 11 11 11 12 11 11 11 11 22 11 11 11 11 11 12 11 11 11 11 11 11 11

B07_north_of_Purbeck_hills/2009_424, 11 11 11 11 12 11 12 11 11 11 11 11 11 11 11 11 11 11 11 11 11 11 11 22 11 12 11 11 11 11 12

B07_north_of_Purbeck_hills/2009_426, 12 11 11 11 11 -9 22 11 11 -9 11 -9 11 11 11 11 11 -9 11 -9 11 11 11 -9 11 11 11 11 -9 -9 -9

B07_north_of_Purbeck_hills/2009_428, 11 11 12 12 12 11 12 12 11 11 11 11 11 11 11 11 12 11 22 11 -9 11 11 12 12 12 11 11 11 11 11

B07_north_of_Purbeck_hills/2009_432, 12 11 12 12 22 11 12 11 11 11 11 11 11 12 11 11 12 11 11 11 11 11 11 12 11 11 11 11 11 11 22

B07_north_of_Purbeck_hills/2009_435, 11 11 11 11 11 11 11 12 12 11 11 12 11 11 11 11 12 11 11 11 11 11 11 12 11 12 11 11 11 11 22

B07_north_of_Purbeck_hills/2009_438, 11 -9 11 11 11 -9 -9 11 12 11 11 11 11 11 11 -9 11 11 11 11 -9 22 11 -9 11 -9 11 11 11 11 12

B07_north_of_Purbeck_hills/2009_443, 12 11 11 12 12 11 22 11 11 11 11 11 12 11 12 11 11 12 11 11 11 11 11 11 11 11 11 12 11 11 11

B07_north_of_Purbeck_hills/2009_444, 11 11 12 11 22 11 12 12 12 11 11 11 11 11 11 11 -9 11 11 11 11 11 11 22 11 11 11 11 11 11 11

B07_north_of_Purbeck_hills/2009_445, 12 11 11 11 22 11 12 11 11 11 11 11 12 11 11 11 11 11 11 11 11 11 11 22 12 11 11 11 11 11 11

B07_north_of_Purbeck_hills/2009_446, 11 12 11 11 12 11 12 11 11 11 11 11 11 11 12 12 12 11 11 11 11 11 11 22 -9 11 11 11 11 11 12

B07_north_of_Purbeck_hills/2009_447, 12 11 11 11 11 11 11 11 11 11 11 11 12 11 11 11 11 12 11 11 11 11 11 12 12 11 11 11 11 11 11

B07_north_of_Purbeck_hills/2009_448, 11 11 12 12 11 11 11 11 11 -9 11 11 11 11 11 11 11 11 11 11 11 11 11 12 11 11 11 11 11 11 12

B07_north_of_Purbeck_hills/2009_449, 11 12 12 11 12 11 11 11 11 11 11 11 22 11 11 11 11 11 11 11 11 11 -9 12 12 22 11 12 -9 11 11

B07_north_of_Purbeck_hills/2009_450, 11 11 11 12 11 11 12 11 11 11 11 11 11 11 11 11 11 11 11 11 11 11 11 22 12 12 11 11 11 11 11

B07_north_of_Purbeck_hills/2009_451, 12 -9 11 12 12 11 11 11 11 11 12 11 11 11 11 11 12 11 11 11 11 11 11 11 11 11 11 11 11 11 12

B07_north_of_Purbeck_hills/2009_452, 11 11 11 12 12 11 12 11 11 12 11 11 12 11 12 11 -9 12 11 11 11 11 11 22 12 11 11 11 11 12 11

B07_north_of_Purbeck_hills/2009_453, 11 11 12 11 12 11 12 12 12 12 11 12 11 11 11 11 12 11 11 11 -9 11 11 22 22 11 11 11 11 11 11

B07_north_of_Purbeck_hills/2009_454, 11 11 11 11 11 11 12 11 11 11 11 12 11 11 11 12 11 11 12 11 11 11 11 12 11 11 12 12 11 11 22

B07_north_of_Purbeck_hills/2009_455, 11 11 11 11 11 11 11 11 11 11 11 11 12 11 11 11 12 11 11 11 11 11 11 12 11 11 11 11 11 11 11

B07_north_of_Purbeck_hills/2009_456, 11 11 11 12 12 11 11 11 11 12 11 11 12 11 11 11 12 11 11 11 -9 11 11 22 11 12 11 11 11 11 11

B07_north_of_Purbeck_hills/2009_457, 11 12 11 22 22 11 12 12 11 12 11 11 11 11 12 11 12 11 11 11 11 11 11 12 12 12 11 11 11 11 11

B07_north_of_Purbeck_hills/2009_458, 11 22 11 11 11 12 11 11 11 12 11 11 22 11 11 12 12 11 11 11 11 11 11 12 11 11 11 12 11 11 11

B07_north_of_Purbeck_hills/2009_459, 11 11 11 12 12 11 12 11 11 11 11 11 11 11 11 11 11 11 11 11 11 11 11 11 12 11 11 11 11 11 12

B07_north_of_Purbeck_hills/2009_460, 11 11 11 11 11 12 12 11 11 12 11 11 12 11 11 11 11 11 11 11 11 11 11 22 11 11 11 11 11 11 12

B07_north_of_Purbeck_hills/2009_461, 11 11 11 12 11 11 11 11 11 11 11 11 11 11 11 12 12 11 11 11 11 12 11 12 12 11 11 11 11 11 12

B07_north_of_Purbeck_hills/2009_462, 11 11 11 11 11 11 11 11 11 11 11 11 11 11 12 11 -9 11 11 11 -9 12 11 22 11 11 11 11 11 11 11

B07_north_of_Purbeck_hills/2009_465, 11 11 12 11 11 11 11 11 11 11 11 11 11 11 11 11 12 11 11 11 11 11 11 11 12 11 11 11 11 11 11

B07_north_of_Purbeck_hills/2009_466, 11 11 12 11 11 11 11 12 11 11 11 11 11 11 11 12 12 12 11 11 11 11 11 22 12 11 11 11 11 11 11

B07_north_of_Purbeck_hills/2009_471, 11 11 12 12 22 11 -9 11 11 11 11 11 11 11 12 12 11 12 11 11 11 12 11 11 11 12 11 12 11 11 12

B07_north_of_Purbeck_hills/2009_472, 11 11 11 12 11 11 11 11 11 12 11 11 11 12 11 11 11 11 12 11 11 11 11 12 -9 11 11 12 11 11 11

B07_north_of_Purbeck_hills/2009_475, 11 11 11 11 12 11 12 12 12 11 11 11 11 11 11 11 11 12 11 11 11 12 11 11 11 12 12 11 11 11 11

B07_north_of_Purbeck_hills/2009_477, 11 11 11 22 11 11 11 12 11 11 11 11 11 11 12 11 12 12 11 11 11 11 11 12 11 11 11 11 11 11 11

B07_north_of_Purbeck_hills/2009_479, 11 11 11 11 11 11 -9 11 11 11 12 11 11 11 11 11 11 11 12 11 11 12 11 11 11 11 11 11 11 11 12

B07_north_of_Purbeck_hills/2009_485, 11 11 11 11 11 11 11 11 11 12 11 11 11 11 11 11 12 11 11 11 11 11 11 22 11 11 11 12 11 11 11

B07_north_of_Purbeck_hills/2009_487, 11 11 11 22 11 11 11 11 11 12 11 11 22 11 11 11 12 11 11 11 11 12 11 22 11 11 11 11 11 11 12

B07_north_of_Purbeck_hills/2009_488, 11 11 11 11 11 12 12 11 11 12 11 11 11 11 11 12 11 11 12 11 11 11 11 12 11 12 12 11 11 11 11

B07_north_of_Purbeck_hills/2009_491, 11 12 11 12 11 11 -9 11 11 11 11 11 12 11 11 11 11 12 12 11 11 11 11 11 11 12 11 11 11 11 11

B07_north_of_Purbeck_hills/2009_493, 11 12 11 -9 22 11 12 11 11 12 11 11 11 11 12 11 12 11 12 11 11 11 11 12 11 11 11 11 11 11 12

B07_north_of_Purbeck_hills/2009_495, 12 11 12 12 12 12 11 11 11 11 11 11 12 11 12 11 11 11 11 11 11 11 11 12 11 11 11 11 11 11 11

B07_north_of_Purbeck_hills/2009_496, 11 11 11 11 11 11 12 11 11 11 11 11 11 11 11 11 12 11 11 11 11 11 11 22 11 11 11 11 11 11 12

B07_north_of_Purbeck_hills/2009_497, 12 11 22 11 11 12 12 11 11 11 11 11 11 11 11 11 12 11 11 11 11 11 11 22 11 11 11 -9 -9 11 11

B07_north_of_Purbeck_hills/2009_502, 12 11 12 11 11 11 12 12 11 11 11 11 12 11 11 11 12 12 11 11 11 11 11 22 11 11 11 11 11 11 11

B07_north_of_Purbeck_hills/2009_503, 11 11 11 11 12 11 12 11 11 11 11 12 12 11 11 11 11 11 11 11 11 12 11 12 11 11 11 11 11 11 22

B07_north_of_Purbeck_hills/2009_504, 11 12 11 12 12 11 12 11 11 12 11 12 11 11 11 11 12 11 11 11 11 11 11 12 11 11 11 11 11 11 22

B07_north_of_Purbeck_hills/2009_505, 12 11 11 11 11 11 12 11 11 11 11 11 12 11 12 11 12 11 12 11 11 11 11 12 12 11 11 12 11 11 11

B07_north_of_Purbeck_hills/2009_506, 11 11 11 11 11 11 12 11 11 11 11 12 11 12 11 12 12 11 22 11 11 11 11 11 11 11 11 11 11 11 11

B07_north_of_Purbeck_hills/2009_507, 11 11 -9 12 12 11 11 11 11 12 11 11 11 11 11 11 11 12 12 11 11 11 11 11 22 11 11 11 11 11 11

B07_north_of_Purbeck_hills/2009_508, 11 11 11 12 22 11 11 11 11 11 11 12 11 11 12 11 12 11 12 11 11 11 11 12 11 11 11 11 -9 11 12

B07_north_of_Purbeck_hills/2009_509, 12 11 12 11 11 11 12 11 11 11 11 11 11 11 12 11 12 11 11 11 11 11 11 12 11 22 11 11 11 11 12

B07_north_of_Purbeck_hills/2009_510, 12 11 11 12 11 11 12 11 -9 11 11 12 11 11 -9 11 11 11 12 11 11 11 11 12 11 11 11 11 11 11 -9

B07_north_of_Purbeck_hills/2009_511, 11 11 11 12 12 11 12 11 11 11 11 12 11 11 12 11 12 11 11 11 11 11 11 22 11 12 11 11 11 11 12

B07_north_of_Purbeck_hills/2009_512, 11 11 11 12 12 11 11 12 11 11 12 11 12 11 12 11 12 11 11 11 11 11 11 12 11 11 11 11 11 11 11

B07_north_of_Purbeck_hills/2009_513, 11 22 11 11 11 11 12 11 11 22 11 11 11 11 11 11 -9 11 11 -9 11 11 11 -9 -9 11 11 11 11 11 12

B07_north_of_Purbeck_hills/2009_514, 11 11 11 11 11 11 12 11 12 12 11 11 11 12 12 11 11 11 12 11 11 11 11 12 12 11 11 11 11 11 11

B07_north_of_Purbeck_hills/2009_517, 11 11 11 12 11 11 12 12 11 11 11 11 11 11 11 12 11 11 11 11 11 11 11 12 12 11 11 11 11 11 12

B07_north_of_Purbeck_hills/2009_522, 11 12 11 11 11 11 11 -9 11 11 11 11 12 11 12 11 11 12 11 11 11 11 11 12 12 12 11 11 11 11 22

B07_north_of_Purbeck_hills/2009_525, 11 11 11 11 11 11 11 12 11 11 11 11 11 11 12 11 11 11 11 11 11 11 11 12 12 11 11 11 11 11 11

B07_north_of_Purbeck_hills/2009_526, 12 11 11 11 12 11 11 11 11 11 11 11 12 11 12 11 11 11 11 11 11 11 11 11 12 11 11 12 11 11 11

B07_north_of_Purbeck_hills/2009_530, 11 11 11 12 11 11 11 22 11 12 12 12 12 -9 11 11 11 11 22 11 11 11 11 22 11 12 11 11 11 11 11

B07_north_of_Purbeck_hills/2009_531, 11 11 11 12 12 11 11 11 11 12 11 22 12 11 12 11 12 12 11 11 11 12 11 12 11 11 11 11 11 11 12

B07_north_of_Purbeck_hills/2009_533, 11 12 11 11 11 12 11 11 11 11 11 11 -9 11 11 11 11 11 12 11 11 12 11 -9 12 12 11 11 11 11 11

B07_north_of_Purbeck_hills/2009_537, 11 12 11 -9 -9 11 12 11 11 11 11 12 12 11 12 11 -9 12 11 11 11 11 11 12 11 12 11 11 11 11 12

B07_north_of_Purbeck_hills/2009_538, 11 11 11 11 11 11 12 11 11 11 11 11 11 12 12 11 12 12 11 11 -9 11 11 22 11 12 12 11 11 11 11

B07_north_of_Purbeck_hills/2009_539, 11 11 11 11 11 12 12 11 11 11 12 11 12 11 11 11 11 11 11 11 11 11 11 12 12 11 11 12 11 11 11

B07_north_of_Purbeck_hills/2009_540, 11 12 11 11 11 11 12 11 11 11 11 11 11 11 12 12 12 11 11 11 11 11 11 -9 11 11 11 11 11 11 12

B07_north_of_Purbeck_hills/2009_543, 11 11 11 11 22 11 11 11 11 11 11 11 11 11 11 11 11 11 11 11 11 11 11 12 12 11 11 12 11 11 12

B07_north_of_Purbeck_hills/2009_545, 11 11 11 12 11 11 12 11 11 12 11 12 11 11 12 11 12 12 11 11 11 11 11 22 11 11 11 11 11 11 12

B07_north_of_Purbeck_hills/2009_554, 11 12 11 11 11 11 11 11 11 11 11 11 11 11 12 11 11 11 11 11 11 11 11 12 11 11 11 11 11 11 11

B07_north_of_Purbeck_hills/2009_555, 11 11 11 11 11 11 11 11 11 12 11 11 11 12 11 11 22 11 11 11 11 11 11 11 11 11 11 11 11 11 11

B07_north_of_Purbeck_hills/2009_556, 11 11 11 11 11 11 22 22 11 12 11 11 12 11 11 11 -9 11 11 11 -9 11 11 22 11 12 11 11 11 11 -9

B07_north_of_Purbeck_hills/2009_561, 11 12 11 11 11 11 11 11 11 11 11 12 12 11 11 11 11 11 11 11 11 11 11 12 22 11 12 11 11 11 11

B07_north_of_Purbeck_hills/2009_563, 11 11 11 11 11 11 12 11 11 12 11 11 12 11 11 11 11 11 11 11 -9 11 11 -9 11 11 11 11 11 11 12

B07_north_of_Purbeck_hills/2009_564a, 11 11 12 11 11 11 11 12 11 11 11 12 11 11 11 12 11 11 11 11 -9 12 11 12 11 11 11 11 11 11 11

B07_north_of_Purbeck_hills/2009_564b, 11 11 11 11 12 11 12 11 11 11 11 12 12 11 11 11 11 11 11 11 -9 11 11 22 11 12 11 11 11 11 12

B07_north_of_Purbeck_hills/2009_570, 11 11 11 11 22 11 11 11 11 11 11 11 11 11 11 11 11 11 11 11 11 11 11 22 12 11 11 11 11 11 11

B07_north_of_Purbeck_hills/2009_593, 11 11 11 11 -9 11 11 11 11 11 11 11 11 11 11 11 11 12 11 11 11 -9 11 11 12 11 11 11 11 11 11

B07_north_of_Purbeck_hills/2009_594, 11 22 12 11 12 11 11 11 11 11 11 11 12 11 12 11 11 11 12 11 11 11 11 22 12 11 11 11 11 11 -9

B07_north_of_Purbeck_hills/2009_595, 11 11 12 12 12 11 11 11 11 12 11 11 11 11 11 11 -9 11 11 11 11 11 11 11 11 11 11 11 11 11 11

B07_north_of_Purbeck_hills/2009_624, 12 11 11 12 11 11 11 11 11 11 11 12 11 11 11 11 12 11 11 11 -9 11 11 22 11 12 11 11 11 11 11

B07_north_of_Purbeck_hills/2009_626, 11 12 11 12 11 11 11 12 11 12 11 11 11 11 11 12 11 11 11 11 -9 11 11 12 11 11 11 11 11 11 12

B07_north_of_Purbeck_hills/2009_627, 11 11 12 11 11 11 12 -9 11 11 11 11 11 11 11 12 12 11 12 -9 11 11 11 12 11 11 11 12 11 11 11

B07_north_of_Purbeck_hills/2009_664, 11 11 11 11 11 11 11 11 12 11 11 12 12 11 11 11 12 11 11 11 11 12 11 11 12 11 11 11 11 11 11

B08_Verwood/4907, 11 12 11 12 12 11 11 11 11 11 11 12 11 11 11 11 11 12 11 11 11 11 11 -9 11 11 11 11 12 11 12

B08_Verwood/4909, -9 -9 -9 11 11 11 11 11 11 11 11 11 11 11 11 11 12 11 11 11 11 12 11 12 11 11 11 11 11 11 12

B08_Verwood/4904, 11 11 11 11 12 11 11 11 11 11 11 11 12 11 12 11 11 11 11 11 11 12 11 -9 11 11 11 11 12 11 11

B08_Verwood/4905, 11 11 11 11 12 11 11 11 11 11 11 11 11 11 11 12 11 11 12 -9 11 11 11 -9 11 11 11 11 11 11 11

B08_Verwood/4906, 11 11 11 11 11 11 11 11 11 11 11 11 11 11 11 12 11 11 11 11 11 11 11 12 11 11 11 11 11 11 11

B08_Verwood/4908, 11 12 11 -9 11 11 11 11 11 11 11 11 11 11 11 12 12 11 12 11 11 11 11 22 12 11 11 11 11 11 11

B09_Mendip/4967, -9 12 12 11 22 12 11 11 11 11 11 12 11 11 11 11 11 11 11 11 11 11 11 12 11 11 11 11 12 11 12

B09_Mendip/4968, 11 12 11 11 12 11 11 11 11 11 11 11 11 11 11 11 12 11 11 11 11 11 11 22 11 11 11 11 11 11 12

B09_Mendip_Priddy_pool/4971, 11 11 11 11 11 11 11 11 11 11 11 11 11 12 11 12 12 11 11 -9 11 11 11 22 22 11 11 11 11 11 11

B09_Mendip_Priddy_pool/4972, 12 11 11 11 11 11 11 11 11 11 11 11 11 11 11 12 11 11 11 11 11 11 11 11 11 11 11 11 22 11 11

B09_Mendip_Waldegrave_pool/4975, 11 11 11 -9 11 -9 -9 11 11 11 -9 11 11 -9 11 11 12 11 11 11 -9 11 -9 -9 12 11 11 11 11 11 -9

B09_Mendip_Waldegrave_pool/4978, 11 11 11 11 12 11 11 11 11 11 11 11 11 12 11 11 12 11 11 11 11 11 11 22 11 11 11 11 11 11 22

B09_Mendip_Waldegrave_pool/4973, 12 11 11 11 11 12 22 11 11 11 11 12 11 11 11 11 12 11 11 11 11 11 11 12 11 11 11 11 12 11 11

B09_Mendip_Waldegrave_pool/4974, 12 11 12 11 11 11 11 11 11 11 11 11 11 11 11 11 12 11 11 11 11 11 11 11 12 11 11 11 11 11 11

B09_Mendip_Waldegrave_pool/4976, -9 11 11 12 22 22 11 11 11 11 11 11 11 11 11 11 11 11 11 11 11 11 11 11 11 12 11 11 12 11 11

B09_Mendip_Waldegrave_pool/4977, 12 12 12 11 11 11 11 11 11 11 11 11 11 11 11 11 12 11 12 11 11 11 11 -9 12 11 11 11 11 11 11

B09_Mendip_Waldegrave_pool/4979, 11 12 11 11 11 11 11 12 11 11 11 -9 12 11 11 11 12 11 11 11 11 11 11 11 12 11 11 11 12 11 11

B09_Mendip_Waldegrave_pool/4980, 11 11 12 11 11 11 11 11 11 11 11 11 11 11 11 11 11 11 12 11 11 11 11 12 11 11 11 11 22 11 11

B10_Salisbury_Plain_cluster/4981, 11 11 11 11 11 11 11 11 11 11 11 11 11 11 11 11 12 11 11 11 11 12 11 12 22 11 11 11 11 11 12

B10_Salisbury_Plain_cluster/4984, 11 11 22 12 12 11 11 11 11 11 11 12 11 11 11 11 11 11 11 11 11 11 11 12 22 11 11 11 11 11 12

B10_Salisbury_Plain_cluster/4982, 11 12 12 11 12 12 11 11 11 11 12 -9 11 11 12 11 11 11 11 11 11 12 11 11 11 11 11 11 11 12 11

B10_Salisbury_Plain_cluster/4983, 11 11 11 11 11 12 11 11 11 11 11 11 12 11 11 11 11 11 11 11 11 11 11 22 22 12 11 12 12 11 11

B10_Salisbury_Plain_cluster/4985, 11 11 11 12 11 11 11 11 11 11 11 11 11 11 11 11 11 11 11 11 11 11 11 22 11 11 11 11 12 11 11

B10_Salisbury_Plain_cluster/4986, 11 11 11 11 11 11 11 11 11 11 11 11 11 11 12 11 12 11 12 11 11 11 11 22 11 11 11 11 11 11 11

B10_Salisbury_Plain_cluster/4987, 11 12 11 11 11 -9 11 11 11 11 11 11 11 12 12 11 11 11 11 11 11 11 11 12 11 11 11 11 11 11 11

B11_Cadeby_Quarry/1644, 11 11 11 11 11 11 11 11 11 11 11 -9 11 11 11 11 11 11 11 11 11 11 11 12 11 11 11 11 11 11 11

B11_Cadeby_Quarry/1645, -9 11 11 -9 12 11 -9 11 11 11 11 11 11 11 11 11 11 11 11 11 11 11 11 12 11 11 11 11 11 11 11

B11_Bagworth_Park/1620, 11 11 11 11 11 11 11 11 11 11 11 11 11 11 11 11 11 11 11 11 -9 11 11 12 11 11 11 11 12 11 11

B11_Bagworth_Park/1621, 11 11 11 -9 11 11 12 11 11 11 11 11 11 11 11 11 11 11 11 11 11 11 -9 12 11 11 11 11 12 11 11

B11_BagworthPark/01, 11 11 11 11 11 11 11 11 11 11 11 11 11 11 11 11 22 11 11 11 11 11 11 12 11 11 11 11 11 11 11

B11_BagworthPark/03, 11 11 11 11 11 11 11 11 11 11 11 11 11 11 11 11 22 11 11 11 11 11 11 22 11 11 11 11 11 11 11

B11_BagworthPark/04, 11 11 11 11 11 11 11 11 11 11 11 11 11 11 11 11 22 11 11 11 11 11 11 11 11 11 11 11 11 11 11

B11_BagworthPark/05, 11 11 11 11 11 11 11 11 11 11 11 11 11 11 12 11 22 11 11 11 11 11 11 12 11 11 11 11 11 11 11

B11_BagworthPark/06, 11 11 11 11 11 11 11 11 11 11 11 11 11 11 11 11 22 11 11 11 11 11 11 12 11 11 11 11 11 11 11

B11_BagworthPark/07, 11 11 11 11 11 11 11 11 11 11 12 11 11 11 11 11 22 11 11 11 11 11 11 11 11 11 11 11 11 11 11

B11_BagworthPark/08, 11 12 11 11 12 11 11 11 11 11 11 11 11 11 22 11 22 11 11 11 11 11 11 11 11 11 11 11 11 11 11

B11_BagworthPark/09, 12 11 11 12 11 11 11 11 11 11 11 11 11 11 11 11 22 11 11 11 11 11 11 22 11 11 11 11 11 11 11

B11_BagworthPark/10, 11 11 11 11 11 11 11 11 11 11 11 11 11 11 11 11 22 11 11 11 11 11 11 12 11 11 11 11 12 11 11

B11_BagworthPark/11, 11 11 11 11 11 11 11 11 11 11 11 11 11 11 11 11 22 11 11 11 11 11 11 22 11 11 11 11 11 11 11

B11_BagworthPark/12, 11 11 11 11 11 11 11 11 11 11 11 11 11 11 11 11 22 11 11 11 11 11 11 12 11 11 11 11 11 11 11

B11_BagworthPark/13, 11 11 11 11 11 11 11 11 11 11 11 11 11 11 12 11 22 11 11 11 11 11 11 12 22 11 11 11 11 11 11

B11_BagworthPark/15, 11 11 11 11 11 11 11 11 11 11 12 11 11 11 11 11 11 11 11 11 11 11 11 12 11 11 11 11 11 11 11

B11_BagworthPark/16, 11 11 11 11 11 11 11 11 11 11 11 11 11 11 11 11 22 11 11 11 11 11 11 22 11 11 11 11 12 11 11

B11_BagworthPark/17, 11 11 11 11 11 11 11 11 11 11 11 11 11 11 11 11 22 11 11 11 11 11 11 11 11 11 11 11 11 11 11

B11_BagworthPark/18, 11 11 11 11 11 11 11 11 11 11 11 11 11 11 11 11 22 11 11 11 11 11 11 12 11 11 11 11 11 11 12

B11_BagworthPark/20, 11 11 11 11 11 11 11 11 11 11 11 11 11 11 11 11 22 11 11 11 11 11 11 12 11 11 11 11 11 11 11

B11_CadebyQuarry/01, 11 11 11 11 11 11 11 11 11 11 11 11 11 11 12 11 22 11 11 11 11 11 11 11 11 11 11 11 11 11 11

B11_CadebyQuarry/02, 12 11 11 11 11 11 11 11 11 11 11 11 11 11 11 11 22 11 11 11 11 11 11 22 11 11 11 11 11 11 11

B11_CadebyQuarry/03, 11 11 11 11 11 11 11 11 11 11 11 11 11 11 11 11 22 11 11 11 11 11 11 22 11 11 11 11 11 11 11

B11_CadebyQuarry/10, 11 11 11 11 12 11 11 11 11 11 11 11 11 11 11 11 22 11 11 11 11 11 11 12 11 11 11 11 11 11 11

B11_CadebyQuarry/11, 11 11 11 11 12 11 11 11 11 11 11 11 11 11 12 11 22 11 11 12 11 11 11 12 11 11 11 11 11 11 11

B11_CadebyQuarry/12, 11 11 11 11 11 11 11 11 11 11 11 11 -9 11 12 11 12 11 11 11 11 11 11 12 11 11 11 11 11 11 11

B11_CadebyQuarry/13, 11 11 11 11 11 11 11 11 11 11 11 11 11 11 11 11 22 11 11 11 11 11 11 12 11 11 11 11 11 11 11

B11_CadebyQuarry/14, 11 11 11 11 12 11 11 11 11 11 11 11 11 11 11 11 22 11 11 11 11 11 11 12 11 11 11 11 11 11 11

B11_CadebyQuarry/15, 12 11 11 11 11 11 11 11 11 11 11 11 11 11 11 11 12 11 11 11 11 11 11 12 11 11 11 11 11 11 11

B11_CadebyQuarry/17, 11 11 11 11 12 11 11 11 11 11 11 11 11 11 11 11 22 11 11 11 11 11 11 12 11 11 11 11 11 11 11

B12_FishPond/1612, 11 11 11 11 11 11 11 11 11 12 11 11 11 11 11 11 11 11 11 11 11 11 11 12 11 11 11 11 11 11 11

B12_Shepshed/1628, 11 11 11 11 11 11 11 11 11 11 11 11 11 11 11 11 11 11 11 11 11 11 11 12 11 11 11 11 11 11 11

B12_Shepshed/1629, 11 11 11 11 11 11 11 11 11 11 11 11 11 11 11 11 11 11 11 11 11 11 11 22 11 11 11 11 11 11 11

B12_FishPond/01, 11 11 11 11 11 11 11 11 11 11 11 11 11 11 11 11 22 11 11 11 11 11 11 12 11 11 11 11 11 11 11

B12_FishPond/02, 11 11 11 11 11 11 11 11 11 11 11 11 11 12 11 11 12 11 11 11 11 11 11 12 11 11 11 11 11 11 11

B12_FishPond/03, 11 11 11 11 12 11 11 11 11 11 11 11 11 11 11 11 22 11 11 11 11 11 11 12 11 11 11 11 11 11 11

B12_FishPond/04, 11 11 11 11 12 11 11 11 11 11 11 11 11 11 11 11 22 11 11 11 11 11 11 22 11 11 11 11 11 11 11

B12_FishPond/05, 11 11 11 11 11 11 11 11 11 11 11 11 11 12 12 11 12 11 11 11 11 11 11 12 11 11 11 11 11 11 11

B12_FishPond/06, 11 11 -9 11 11 11 11 11 11 11 11 11 11 11 11 11 22 11 11 11 11 11 11 12 11 11 11 11 11 11 11

B12_FishPond/07, 11 11 11 11 11 11 11 11 11 11 11 11 11 12 12 11 22 11 11 11 11 11 11 12 11 11 11 11 11 11 11

B12_FishPond/08, 11 11 11 11 12 11 11 11 11 11 11 11 11 11 12 11 22 11 11 11 11 11 11 12 11 11 11 11 11 11 11

B12_FishPond/11, 11 11 11 11 12 11 11 11 11 11 11 11 11 11 11 11 22 11 11 11 11 11 11 11 11 11 11 11 11 11 11

B12_FishPond/14, 11 11 11 11 11 11 11 11 11 11 11 11 11 11 11 11 22 11 11 11 11 11 11 12 11 11 11 11 11 11 11

B12_FishPond/15, 11 11 11 11 11 11 11 11 11 11 11 11 11 11 11 11 22 11 12 11 11 11 11 11 11 11 11 11 11 11 11

B12_Shepshed/03, 11 11 11 11 11 11 11 11 11 11 11 11 11 11 11 11 22 11 11 11 11 11 11 12 11 11 11 11 11 11 11

B12_Shepshed/04, 11 11 11 11 11 11 11 11 11 11 11 11 11 11 11 11 22 11 11 11 11 11 11 11 11 11 11 11 11 11 11

B12_Shepshed/05, 11 11 11 11 11 11 11 11 11 11 11 11 11 11 11 11 22 11 11 11 11 11 11 12 11 11 11 11 11 11 11

B12_Shepshed/06, 11 11 11 11 11 11 11 11 11 11 11 11 11 11 11 11 22 11 11 11 11 11 11 12 11 11 11 11 11 11 11

B12_Shepshed/07, 11 11 11 12 11 11 11 11 11 11 11 11 11 11 12 11 22 11 11 11 11 11 11 22 11 11 11 11 11 11 11

B12_Shepshed/09, 11 11 11 11 11 11 11 11 11 11 11 11 11 11 11 11 22 11 11 11 11 11 11 11 11 11 11 11 11 11 11

B12_Shepshed/12, 11 11 11 11 11 11 11 11 11 11 11 11 11 11 11 11 22 11 11 11 11 11 11 11 11 11 11 12 11 11 11

B12_Shepshed/14, 11 11 11 11 11 11 11 11 11 11 11 11 11 11 11 11 22 11 11 11 11 11 11 12 12 12 11 11 11 11 11

B12_Shepshed/16, 11 11 11 11 11 11 12 11 11 11 11 11 11 11 11 11 12 11 11 11 11 11 11 12 11 11 11 11 11 11 11

B13_Braunston/1636, -9 11 11 11 11 11 11 11 11 11 11 11 11 11 11 11 11 11 11 11 11 -9 11 11 -9 11 11 11 11 11 11

B13_Braunston/1637, 11 11 11 11 11 11 11 11 11 11 11 11 11 11 11 11 11 11 11 11 11 11 11 22 11 11 11 11 11 11 11

B14_Illington/4922, 11 11 11 11 11 11 11 11 11 11 11 11 11 11 11 11 -9 11 11 -9 11 11 11 11 11 11 11 11 11 11 11

B14_Illington/4923, 11 11 11 11 11 11 11 11 11 11 11 11 11 11 11 11 11 11 11 11 11 11 11 -9 11 11 11 11 11 11 11

B14_Illington/4924, 11 11 11 11 11 11 11 11 11 11 11 11 11 11 11 11 11 11 11 11 11 11 11 -9 11 11 11 11 11 11 11

B14_Illington/4925, 11 11 11 11 11 11 -9 11 11 11 11 11 11 11 11 11 11 11 11 11 11 11 11 -9 11 11 11 11 11 11 11

B14_Illington/4926, 11 11 11 11 11 11 11 11 11 11 11 11 11 11 11 11 11 11 11 11 11 11 11 11 11 11 11 11 11 11 11

B14_Illington/4927, 11 11 11 11 11 11 11 11 11 11 11 11 11 11 11 11 11 11 11 11 11 11 11 12 11 11 11 11 11 11 11

B14_Illington/4928, 11 11 11 11 11 11 11 11 11 11 11 11 11 11 11 11 11 11 11 11 11 11 11 -9 11 11 11 11 11 11 11

B14_Illington/4929, 11 11 11 11 11 11 11 11 11 11 11 11 11 11 11 11 11 11 11 11 11 11 11 12 11 11 11 11 11 11 11

B15_Loch_na_Crann_Highland/4988, 11 11 11 11 11 11 11 11 11 11 11 11 11 11 11 11 11 11 11 11 11 11 11 11 11 11 11 11 11 11 11

B15_Loch_na_Crann_Highland/4989, 11 11 11 11 11 11 11 11 11 11 11 11 11 11 11 11 11 11 11 11 11 11 11 11 11 11 11 11 11 11 11

B15_Loch_na_Crann_Highland/4990, 11 11 11 11 11 11 11 11 11 11 11 11 11 11 11 11 12 11 11 11 11 12 11 12 11 11 11 11 11 11 11

B15_Loch_na_Crann_Highland/4991, 11 11 11 11 11 11 11 11 11 11 11 11 11 11 11 11 11 11 11 11 11 11 11 11 11 11 11 11 11 11 11

B15_Loch_na_Crann_Highland/4992, 11 11 11 11 11 11 11 11 11 11 11 11 11 11 11 11 -9 11 11 11 11 11 11 11 11 11 11 11 11 11 11

B15_Loch_na_Crann_Highland/4993, 11 11 11 11 11 11 11 11 11 11 11 11 11 11 11 11 11 11 11 11 11 11 11 11 11 11 11 11 11 11 11

B15_Loch_na_Crann_Highland/4994, 12 11 11 11 11 11 11 12 11 11 11 11 11 11 11 11 -9 11 11 11 11 11 11 -9 11 11 11 11 11 11 11

B15_Loch_na_Crann_Highland/4995, -9 11 11 11 11 11 11 12 11 11 11 11 11 11 11 11 12 11 11 11 -9 11 11 11 11 11 11 11 11 11 11

N01_A1/01, 11 11 11 11 11 11 11 11 11 11 11 11 11 11 11 11 11 11 11 11 11 11 11 -9 11 11 11 11 -9 11 11

N01_A1/02, 11 11 11 11 11 11 11 11 11 11 11 11 11 11 11 11 11 11 11 11 11 11 11 11 11 11 11 11 11 11 11

N01_A1/03, 11 11 11 11 11 11 11 11 11 11 11 11 11 11 11 11 12 11 11 11 11 11 11 11 11 11 11 11 11 11 11

N01_A1/04, 11 11 11 11 11 11 11 11 11 11 11 11 -9 11 11 11 12 11 11 11 11 11 11 -9 11 11 11 11 -9 11 11

N01_A1/05, 22 11 11 11 11 11 11 11 11 11 11 11 11 11 11 11 11 11 11 11 11 11 11 11 11 11 11 11 11 11 11

N01_A1/06, 22 11 11 11 11 11 -9 11 11 -9 11 -9 11 11 11 11 11 11 11 -9 11 11 11 -9 -9 11 11 11 -9 11 11

N01_A1/07, 22 11 11 11 11 11 11 11 11 11 11 11 11 11 11 11 11 11 11 11 11 -9 11 11 11 11 11 11 11 11 11

N02_A2/04, 12 11 11 11 11 11 11 11 11 11 11 11 11 11 11 11 22 11 11 11 11 11 11 11 11 11 11 11 11 11 11

N02_A2/07, 11 11 11 11 11 11 11 11 11 11 11 11 11 11 11 11 11 11 11 11 11 11 11 11 11 11 11 11 11 11 11

N02_A2/11, 12 11 11 11 11 11 11 11 11 11 11 11 11 11 11 11 22 11 11 11 11 11 11 11 11 11 11 11 11 11 11

N03_A3/01, 11 11 11 11 11 11 11 11 11 11 11 11 -9 11 11 11 22 11 11 11 11 11 11 -9 11 11 11 11 -9 11 11

N03_A3/04, 11 11 11 11 11 11 11 11 11 11 11 11 11 11 11 11 11 11 11 11 11 11 11 11 11 11 11 11 11 11 11

N03_A3/05, 11 11 11 11 11 11 11 11 11 -9 11 11 11 11 11 11 22 11 11 -9 11 11 11 11 -9 11 11 11 11 11 11

N03_A3/06, 11 11 11 11 11 11 11 11 11 11 11 11 11 11 11 11 12 11 11 11 11 11 11 11 11 11 11 11 11 11 11

N03_A3/07, 11 11 11 11 11 11 11 11 11 11 11 11 11 11 11 11 22 11 11 11 11 11 11 11 11 11 11 11 11 11 11

N03_A3/08, 11 11 11 11 11 11 11 11 11 11 11 11 11 11 11 11 22 11 11 11 11 11 11 11 11 11 11 11 11 11 11

N03_A3/09, 12 11 11 11 11 11 11 11 11 11 11 11 11 11 11 11 12 11 11 11 11 11 11 11 11 11 11 11 11 11 11

N03_A3/12, 11 11 11 11 11 11 11 11 11 11 11 11 11 11 11 11 22 11 11 11 11 11 11 11 11 11 11 11 11 11 11

N03_A3/13, 12 11 11 11 11 11 11 11 11 11 11 11 11 11 11 11 22 11 11 11 11 11 11 11 11 11 11 11 11 11 11

N03_A3/14, 11 11 11 11 11 11 11 11 11 11 11 11 11 11 11 11 22 11 11 11 11 11 11 11 11 11 11 11 11 11 11

N03_A3/15, 22 11 11 11 11 11 11 11 11 11 11 11 11 11 11 11 12 11 11 11 11 11 11 11 11 11 11 11 11 11 11

N03_A3/16, 11 11 11 11 11 11 11 11 11 11 11 11 11 11 11 11 12 11 11 11 11 11 11 11 11 11 11 11 11 11 11

N03_A3/17, 11 11 11 11 11 11 11 11 11 11 11 11 11 11 11 11 12 11 11 11 11 11 11 11 11 11 11 11 11 11 11

N03_A3/18, 12 11 11 11 11 11 11 11 11 11 11 11 11 11 11 11 22 11 11 11 11 11 11 11 11 11 11 11 11 11 11

N03_A3/19, 12 11 11 11 11 11 11 11 11 11 11 11 11 11 11 11 22 11 11 11 11 11 11 11 11 11 11 11 11 11 11

N03_A3/20, 12 11 11 11 11 11 11 11 11 11 11 11 11 11 11 11 12 11 11 11 11 11 11 11 11 11 11 11 11 11 11

N03_A3/21, 22 11 11 11 11 11 11 11 11 11 11 11 11 11 11 11 22 11 11 11 11 11 11 11 11 11 11 11 11 11 11

N03_A3/22, 11 11 11 11 11 11 11 11 11 11 11 11 11 11 11 11 22 11 11 11 11 11 11 11 11 11 11 11 11 11 11

N04_B1/01, 22 11 11 11 11 11 11 11 11 11 11 11 11 11 11 11 12 11 11 11 11 11 11 11 11 11 11 11 11 11 11

N04_B1/02, 12 11 11 11 11 11 11 11 11 11 11 11 -9 11 11 11 11 11 11 11 11 11 11 11 11 11 11 11 11 11 11

N04_B1/03, 12 11 11 11 11 11 11 11 11 11 11 11 11 11 11 11 11 11 11 11 11 11 11 11 11 11 11 11 11 11 11

N04_B1/04, 22 11 11 11 11 11 11 11 11 11 11 11 11 11 11 11 11 11 11 11 11 11 11 11 11 11 11 11 11 11 11

N04_B1/05, 22 11 11 11 11 11 11 11 11 11 11 11 11 11 11 11 11 11 11 11 11 11 11 11 11 11 11 11 11 11 11

N05_B2/01, 22 11 11 11 11 11 11 11 11 11 11 11 11 11 11 11 11 11 11 11 11 11 11 11 11 11 11 11 11 11 11

N05_B2/02, 22 11 11 11 11 11 11 11 11 11 11 11 11 11 11 11 11 11 11 11 11 11 11 11 11 11 11 11 11 11 11

N05_B2/03, 22 11 11 11 11 11 11 11 11 11 11 11 11 11 11 11 12 11 11 11 11 11 11 11 11 11 11 11 11 11 11

N05_B2/04, 22 11 11 11 11 11 11 11 11 11 11 11 11 11 11 11 11 11 11 11 11 11 11 11 11 11 11 11 11 11 11

N05_B2/05, 22 11 11 11 11 11 11 11 11 11 11 11 11 11 11 11 12 11 11 11 11 11 11 11 11 11 11 11 11 11 11

N05_B2/06, 22 11 11 11 11 11 11 11 11 11 11 11 11 11 11 11 11 11 11 11 11 11 11 11 11 11 11 11 11 11 11

N05_B2/07, 22 11 11 11 11 11 11 11 11 11 11 11 11 11 11 11 11 11 11 11 11 11 11 11 11 11 11 11 11 11 11

N06_B4/01, 11 11 11 11 11 11 11 11 11 11 11 11 11 11 11 11 12 11 11 11 11 11 11 11 11 11 11 11 11 11 11

N06_B4/02, 22 11 11 11 11 11 11 11 11 11 11 11 11 11 11 11 11 11 11 11 11 11 11 11 11 11 11 11 11 11 11

N06_B4/03, 11 11 11 11 11 11 11 11 11 11 11 11 11 11 11 11 22 11 11 11 11 11 11 11 11 11 11 11 11 11 11

N06_B4/04, 11 11 11 11 11 11 11 11 11 11 11 11 11 11 11 11 12 11 11 11 11 11 11 11 11 11 11 11 11 11 11

N06_B4/05, 12 11 11 11 11 11 11 11 11 11 11 11 11 11 11 11 12 11 11 11 11 11 11 11 11 11 11 11 11 11 11

N06_B4/06, 11 11 11 11 11 11 11 11 11 11 11 11 11 11 11 11 11 11 11 11 11 11 11 11 11 11 11 11 11 11 11

N06_B4/07, 11 11 11 11 11 11 11 11 11 11 11 11 11 11 11 11 22 11 11 11 11 11 11 11 11 11 11 11 11 11 11

N07_B5/01, 22 11 11 11 11 11 11 11 11 11 11 11 11 11 11 11 12 11 11 11 11 11 11 11 11 11 11 11 11 11 11

N07_B5/02, 22 11 11 11 11 11 11 11 11 11 11 11 11 11 11 11 12 11 11 11 11 11 11 11 11 11 11 11 11 11 11

N07_B5/03, 22 11 11 11 11 11 11 11 11 11 11 11 11 11 11 11 12 11 11 11 11 11 11 11 11 11 11 11 11 11 11

N07_B5/04, 22 11 11 11 11 11 11 11 11 11 11 11 11 11 11 11 11 11 11 11 11 11 11 11 11 11 11 11 11 11 11

N07_B5/05, 22 11 11 11 11 11 11 11 11 11 11 11 11 11 11 11 12 11 11 11 11 11 11 11 11 11 11 11 11 11 11

N07_B5/06, 22 11 11 11 11 11 11 11 11 11 11 11 11 11 11 11 12 11 11 11 11 11 11 11 11 11 11 11 11 11 11

N07_B5/07, 22 11 11 11 11 11 11 11 11 11 11 11 11 11 11 11 12 11 11 11 11 11 11 11 11 11 11 11 11 11 11

N07_B5/08, 22 11 11 11 11 11 11 11 11 11 11 11 11 11 11 11 11 11 11 11 11 11 11 11 11 11 11 11 11 11 11

N07_B5/09, 22 11 11 11 11 11 11 11 11 11 11 11 11 11 11 11 12 11 11 11 11 11 11 11 11 11 11 11 11 11 11

N07_B5/10, 22 11 11 11 11 11 11 11 11 11 11 11 11 11 11 11 12 11 11 11 11 11 11 11 11 11 11 11 11 11 11

N07_B5/11, 22 11 11 11 11 11 11 11 11 11 11 11 11 11 11 11 22 11 11 11 11 11 11 11 11 11 11 11 11 11 11

N07_B5/12, 22 11 11 11 11 11 11 11 11 11 11 11 11 11 11 11 12 11 11 11 11 11 11 11 11 11 11 11 11 11 11

N07_B5/13, 12 11 11 11 11 11 11 11 11 11 11 11 11 11 11 11 12 11 11 11 11 11 11 11 11 11 11 11 11 11 11

N07_B5/14, 22 11 11 11 11 11 11 11 11 11 11 11 11 11 11 11 22 11 11 11 11 11 11 11 11 11 11 11 11 11 11

N07_B5/15, 22 11 11 11 11 11 11 11 11 11 11 11 11 11 11 11 11 11 11 11 11 11 11 11 11 11 11 11 11 11 11

N07_B5/16, 22 11 11 11 11 11 11 11 11 11 11 11 11 11 11 11 11 11 11 11 11 11 11 11 -9 11 11 11 11 11 11

N07_B5/17, 22 11 11 11 11 11 11 11 11 11 11 11 11 11 11 11 11 11 11 11 11 11 11 11 11 11 11 11 11 11 11

N07_B5/18, 22 11 11 11 11 11 11 11 11 11 11 11 11 11 11 11 11 11 11 11 11 11 11 11 11 11 11 11 11 11 11

N07_B5/19, 22 11 11 11 11 11 11 11 11 11 11 11 11 11 11 11 11 11 11 11 11 11 11 11 11 11 11 11 11 11 11

N07_B5/20, 22 11 11 11 11 11 11 11 11 11 11 11 11 11 11 11 11 11 11 11 11 11 11 11 11 11 11 11 11 11 11

N07_B5/21, 22 11 11 11 11 11 11 11 11 11 11 11 11 11 11 11 11 11 11 11 11 11 11 11 11 11 11 11 11 11 11

N07_B5/22, 22 11 11 11 11 11 11 11 11 11 11 11 11 11 11 11 12 11 11 11 11 11 11 11 11 11 11 11 11 11 11

N07_B5/23, 22 11 11 11 11 11 11 11 11 11 11 11 11 11 11 11 11 11 11 11 11 11 11 11 11 11 11 11 11 11 11

N08_FB/01, 22 11 11 11 11 11 11 11 11 11 11 11 11 11 11 11 11 11 11 11 11 11 11 11 11 11 11 11 11 11 11

N08_FB/02, 22 11 11 11 11 11 11 11 11 11 11 11 11 11 11 11 11 11 11 11 11 11 11 11 11 11 11 11 11 11 11

N08_FB/03, 22 11 11 11 11 11 11 11 11 11 11 11 11 11 11 11 12 11 11 11 11 11 11 11 11 11 11 11 11 11 11

N08_FB/04, 22 11 11 11 11 11 11 11 11 11 11 11 11 11 11 11 11 11 11 11 11 11 11 11 11 11 11 11 11 11 11

N08_FB/05, 22 11 11 11 11 11 11 11 11 11 11 11 11 11 11 11 11 11 11 11 11 11 11 11 11 11 11 11 11 11 11

N08_FB/06, 22 11 11 11 11 11 11 11 11 11 11 11 11 11 11 11 11 11 11 11 11 11 11 11 11 11 11 11 11 11 11

N08_FB/07, 22 11 11 11 11 11 11 11 11 11 11 11 11 11 11 11 11 11 11 11 11 11 11 11 11 11 11 11 11 11 11

N08_FB/08, 22 11 11 11 11 11 11 11 11 11 11 11 11 11 11 11 11 11 11 11 11 11 11 11 11 11 11 11 11 11 11

N08_FB/09, 22 11 11 11 11 11 11 11 11 11 11 11 11 11 11 11 11 11 11 11 11 11 11 11 11 11 11 11 11 11 11

N08_FB/10, 22 11 11 11 11 11 11 11 11 11 11 11 11 11 11 11 11 11 11 11 11 11 11 11 11 11 11 11 11 11 11

N08_FB/11, 22 11 11 11 11 11 11 11 11 11 11 11 11 11 11 11 11 11 11 11 11 11 11 11 11 11 11 11 11 11 11

N08_FB/12, 22 11 11 11 11 11 11 11 11 11 11 11 11 11 11 11 11 11 11 11 11 11 11 11 11 11 11 11 11 11 11

N08_FB/13, 22 11 11 11 11 11 11 11 11 11 11 11 11 11 11 11 11 11 11 11 11 11 11 11 11 11 11 11 11 11 11

N08_FB/14, 22 11 11 11 11 11 11 11 11 11 11 11 11 11 11 11 11 11 11 11 11 11 11 11 11 11 11 11 11 11 11

N08_FB/15, 12 11 11 11 11 11 11 11 11 11 11 11 11 11 11 11 11 11 11 11 11 11 11 11 11 11 11 11 11 11 11

N08_FB/16, 22 11 11 11 11 11 11 11 11 11 11 11 11 11 11 11 11 11 11 11 11 11 11 11 11 11 11 11 11 11 11

N08_FB/17, 22 11 11 11 11 11 11 11 11 11 11 11 11 11 11 11 11 11 11 11 11 11 11 11 11 11 11 11 11 11 11

N08_FB/18, 22 11 11 11 11 11 11 11 11 11 11 11 11 11 11 11 11 11 11 11 11 11 11 11 11 11 11 11 11 11 11

N08_FB/19, 22 11 11 11 11 11 11 11 11 11 11 11 11 11 11 11 11 11 11 11 11 11 11 11 11 11 11 11 11 11 11

N08_FB/20, 22 11 11 11 11 11 11 11 11 11 11 11 11 11 11 11 11 11 11 11 11 11 11 11 11 11 11 11 11 11 11

N09_FN/01, 22 11 11 11 11 11 11 11 11 11 11 11 11 11 11 11 11 11 11 11 11 11 11 11 11 11 11 11 11 11 11

N09_FN/02, 12 11 11 11 11 11 11 11 11 11 11 11 11 11 11 11 12 11 11 11 11 11 11 11 11 11 11 11 11 11 11

N09_FN/03, 22 11 11 11 11 11 11 11 11 11 11 11 11 11 11 11 12 11 11 11 11 11 11 11 11 11 11 11 11 11 11

N09_FN/04, 22 11 11 11 11 11 11 11 11 11 11 11 11 11 11 11 11 11 11 11 11 11 11 11 11 11 11 11 11 11 11

N09_FN/05, 11 11 11 11 11 11 11 11 11 11 11 11 11 11 11 11 11 11 11 11 11 11 11 11 11 11 11 11 11 11 11

N09_FN/06, 22 11 11 11 11 11 11 11 11 11 11 11 11 11 11 11 11 11 11 11 11 11 11 11 11 11 11 11 11 11 11

N09_FN/07, 12 11 11 11 11 11 11 11 11 11 11 11 11 11 11 11 11 11 11 11 11 11 11 11 11 11 11 11 11 11 11

N09_FN/08, 12 11 11 11 11 11 11 11 11 11 11 11 11 11 11 11 11 11 11 11 11 11 11 11 11 11 11 11 11 11 11

N09_FN/09, 12 11 11 11 11 11 11 11 11 11 11 11 11 11 11 11 11 11 11 11 11 11 -9 11 11 11 11 11 11 11 11

N09_FN/10, 22 11 11 11 11 11 11 11 11 11 11 11 11 11 11 11 11 11 11 11 11 11 11 11 11 11 11 11 11 11 11

N09_FN/11, 22 11 11 11 11 11 11 11 11 11 11 11 11 11 11 11 11 11 11 11 11 11 11 11 11 11 11 11 11 11 11

N09_FN/12, 22 11 11 11 11 11 11 11 11 11 11 11 11 11 11 11 12 11 11 11 11 11 11 11 11 11 11 11 11 11 11

N09_FN/13, 12 11 11 11 11 11 11 11 11 11 11 11 11 11 11 11 12 11 11 11 11 11 11 11 11 11 11 11 11 11 11

N09_FN/14, 22 11 11 11 11 11 11 11 11 11 11 11 11 11 11 11 22 11 11 11 11 11 11 11 11 11 11 11 11 11 11

N09_FN/15, 12 11 11 11 11 11 11 11 11 11 11 11 11 11 11 11 12 11 11 11 11 11 11 11 11 11 11 11 11 11 11

N09_FN/16, 22 11 11 11 11 11 11 11 11 11 11 11 11 11 11 11 11 11 11 11 11 11 11 11 11 11 11 11 11 11 11

N09_FN/17, 22 11 11 11 11 11 11 11 11 11 11 11 11 11 11 11 11 11 11 11 11 11 11 11 11 11 11 11 11 11 11

N09_FN/18, 22 11 11 11 11 11 11 11 11 11 11 11 11 11 11 11 11 11 11 11 11 11 11 11 11 11 11 11 11 11 11

N09_FN/19, 22 11 11 11 11 11 11 11 11 11 11 11 11 11 11 11 12 11 11 11 11 11 11 11 11 11 11 11 11 11 11

N09_FN/20, 22 11 11 11 11 11 11 11 11 11 11 11 11 11 11 11 12 11 11 11 11 11 11 11 11 11 11 11 11 11 11

N10_FV/01, 22 11 11 11 11 11 11 11 11 11 11 11 11 11 11 11 12 11 11 11 11 11 11 11 11 11 11 11 11 11 11

N10_FV/02, 11 11 11 11 11 11 11 11 11 11 11 11 11 11 11 11 11 11 11 11 11 11 11 11 11 11 11 11 11 11 11

N10_FV/03, 22 11 11 11 11 11 11 11 11 11 11 11 11 11 11 11 11 11 11 11 -9 11 11 11 11 11 11 11 11 11 11

N10_FV/04, 12 11 11 11 11 11 11 11 11 11 11 11 11 11 11 11 11 11 11 11 11 11 11 11 11 11 11 11 11 11 11

N10_FV/05, 12 11 -9 11 11 11 11 11 11 11 11 11 11 11 11 11 11 11 11 11 11 11 11 11 11 11 11 11 11 11 11

N10_FV/06, 11 11 11 11 11 11 11 11 11 11 11 11 11 11 11 11 11 11 11 11 11 11 11 11 11 11 11 11 11 11 11

N10_FV/07, 22 11 11 11 11 11 11 11 11 11 11 11 11 11 11 11 11 11 11 11 11 11 11 11 11 11 11 11 11 11 11

N10_FV/08, 22 11 11 11 11 11 11 11 11 11 11 11 -9 11 11 11 11 11 11 11 11 11 11 11 11 11 11 11 11 11 11

N10_FV/09, 12 11 11 11 11 11 11 11 11 11 11 11 11 11 11 11 11 11 11 11 11 11 11 11 11 11 11 11 11 11 11

N10_FV/10, 12 11 11 11 11 11 11 11 11 11 11 11 11 11 11 11 11 11 11 11 11 11 11 11 11 11 11 11 11 11 11

N10_FV/11, 22 11 11 11 11 11 11 11 11 11 11 11 11 11 11 11 11 11 11 11 11 11 11 11 11 11 11 11 11 11 11

N10_FV/12, 22 11 11 11 11 11 11 11 11 11 11 11 11 11 11 11 11 11 11 11 11 11 11 11 11 11 11 11 11 11 11

N10_FV/13, 22 11 11 11 11 11 11 11 11 11 11 11 11 11 11 11 11 11 11 11 11 11 11 11 11 11 11 11 11 11 11

N10_FV/14, 22 11 11 11 11 11 11 11 11 11 11 11 11 11 11 11 12 11 11 11 11 11 11 11 11 11 11 11 11 11 11

N10_FV/15, 12 11 11 11 11 11 11 11 11 11 11 11 11 11 11 11 11 11 11 11 11 11 11 11 11 11 11 11 11 11 11

N10_FV/16, 11 11 11 11 11 11 11 11 11 11 11 11 11 11 11 11 11 11 11 11 11 11 11 11 11 11 11 11 11 11 11

N10_FV/17, 22 11 11 11 11 11 11 11 11 11 11 11 11 11 11 11 12 11 11 11 11 11 11 11 11 11 11 11 11 11 11

N10_FV/18, 22 11 11 11 11 11 11 11 11 11 11 11 11 11 11 11 11 11 11 11 11 11 11 11 11 11 11 11 11 11 11

N10_FV/19, 22 11 11 11 11 11 11 11 11 11 11 11 11 11 11 11 11 11 11 11 11 11 11 11 11 11 11 11 11 11 11

N10_FV/20, 22 11 11 11 11 11 11 11 11 11 11 11 11 11 11 11 12 11 11 11 11 11 11 11 11 11 11 11 11 11 11

N10_FV/21, 22 11 11 11 11 11 11 11 11 11 11 11 11 11 11 11 11 11 11 11 11 11 11 11 11 11 11 11 11 11 11

N10_FV/22, 22 11 11 11 11 11 11 11 11 11 11 11 11 11 11 11 11 11 11 11 11 11 11 11 11 11 11 11 11 11 11

N10_FV/23, 22 11 11 11 11 11 11 11 11 11 11 11 11 11 11 11 11 11 11 11 11 11 11 11 11 11 11 11 11 11 11

N11_M1/01, 11 11 11 11 11 11 11 11 11 11 11 11 11 11 11 11 11 11 11 11 11 11 11 11 11 11 11 11 11 11 11

N11_M1/02, 11 11 11 11 11 11 11 11 11 11 11 11 11 11 11 11 12 11 11 11 11 11 11 11 11 11 11 11 11 11 11

N11_M1/03, 22 11 11 11 11 11 11 11 11 11 11 11 11 11 11 11 11 11 11 11 11 11 11 11 11 11 11 11 11 11 11

N11_M1/04, 22 11 11 11 11 11 11 11 11 11 11 11 11 11 11 11 11 11 11 11 11 11 11 11 11 11 11 11 11 11 11

N11_M1/05, 12 11 11 11 11 11 11 11 11 11 11 11 11 11 11 11 11 11 11 11 11 11 11 11 11 11 11 11 11 11 11

N11_M1/06, 11 11 11 11 11 11 11 11 11 11 11 11 11 11 11 11 11 11 11 11 11 11 11 11 11 11 11 11 11 11 11

N11_M1/07, 11 11 11 11 11 11 11 11 11 11 11 11 11 11 11 11 12 11 11 11 11 11 11 11 11 11 11 11 11 11 11

N11_M1/08, 22 11 11 11 11 11 11 11 11 11 11 11 11 11 11 11 11 11 11 11 11 11 11 11 11 11 11 11 11 11 11

N11_M1/09, 12 11 11 11 11 11 11 11 11 11 11 11 11 11 11 11 12 11 11 11 11 11 11 11 11 11 11 11 11 11 11

N11_M1/10, 11 11 11 11 11 11 11 11 11 11 11 11 11 11 11 11 12 11 11 11 11 11 22 11 11 11 11 11 11 11 11

N11_M1/11, 12 11 11 11 11 11 11 11 11 11 11 11 11 11 11 11 11 11 11 11 11 11 11 11 11 11 11 11 11 11 11

N11_M1/12, 12 11 11 11 11 11 11 11 11 11 11 11 11 11 11 11 12 11 11 11 11 11 11 11 11 11 11 11 11 11 11

N11_M1/13, 22 11 11 11 11 11 11 11 11 11 11 11 11 11 11 11 11 11 11 11 11 11 11 11 11 11 11 11 11 11 11

N11_M1/14, 11 11 11 11 11 11 11 11 11 11 11 11 11 11 11 11 12 11 11 11 11 11 11 11 11 11 11 11 11 11 11

N11_M1/15, 12 11 11 11 11 11 11 11 11 11 11 11 11 11 11 11 12 11 11 11 11 11 11 11 11 11 11 11 11 11 11

N11_M1/16, 11 11 11 11 11 11 11 11 11 11 11 11 11 11 11 11 12 11 11 11 11 11 11 11 11 11 11 11 11 11 11

N11_M1/17, 11 11 11 11 11 11 11 11 11 11 11 11 11 11 11 11 12 11 11 11 11 11 11 11 11 11 11 11 11 11 11

N11_M1/18, 12 11 11 11 11 11 11 11 11 11 11 11 11 11 11 11 11 11 11 11 11 11 11 11 11 11 11 11 11 11 11

N11_M1/19, 11 11 11 11 11 11 11 11 11 11 11 11 11 11 11 11 11 11 11 11 11 11 11 11 11 11 11 11 11 11 11

N11_M1/20, 22 11 11 11 11 11 11 11 11 -9 11 11 11 11 11 11 11 11 11 11 11 11 11 11 11 11 11 11 11 11 11

N12_M2/01, 22 11 11 11 11 11 11 11 11 11 11 11 11 11 11 11 12 11 11 11 11 11 11 11 11 11 11 11 11 11 11

N12_M2/02, 22 11 11 11 11 11 11 11 11 11 11 11 11 11 11 11 11 11 11 11 11 11 11 11 11 11 11 11 11 11 11

N12_M2/03, 22 11 11 11 11 11 11 11 11 11 11 11 11 11 11 11 12 11 11 11 11 11 11 11 11 11 11 11 11 11 11

N12_M2/04, 12 11 11 11 11 11 11 11 11 11 11 11 11 11 11 11 12 11 11 11 11 11 11 11 11 11 11 11 11 11 11

N12_M2/05, 22 11 11 11 11 11 11 11 11 11 11 11 11 11 11 11 12 11 11 11 11 11 11 11 11 11 11 11 11 11 11

N12_M2/06, 22 -9 11 -9 11 11 -9 11 11 -9 11 -9 11 11 11 11 12 11 11 -9 11 11 -9 11 -9 11 11 11 11 11 -9

N12_M2/07, 22 11 11 11 11 11 11 11 11 11 11 11 11 11 11 11 11 11 11 11 11 11 11 11 11 11 11 11 11 11 11

N12_M2/08, 22 11 11 11 11 11 11 11 11 11 11 11 11 11 11 11 12 11 11 11 11 11 11 11 11 11 11 11 11 11 11

N12_M2/09, 22 11 11 11 11 11 11 11 11 11 11 11 11 11 11 11 11 11 11 11 11 11 11 11 11 11 11 11 11 11 11

N12_M2/10, 22 11 11 11 11 11 -9 11 11 -9 11 11 11 11 11 11 12 11 11 -9 11 11 11 11 -9 11 11 11 11 11 11

N12_M2/11, 12 11 11 11 11 11 11 11 11 11 11 11 11 11 11 11 11 11 11 11 11 11 11 11 11 11 11 11 11 11 11

N12_M2/12, 11 11 11 11 11 11 11 11 11 11 11 11 11 11 11 11 22 11 11 11 11 11 11 11 11 11 11 11 11 11 11

N12_M2/13, 22 11 11 11 11 11 11 11 11 11 11 11 11 11 11 11 11 11 11 11 11 11 11 11 11 11 11 11 11 11 11

N12_M2/14, 22 11 11 11 11 11 11 11 11 11 11 11 11 11 11 11 11 11 11 11 11 11 11 11 11 11 11 11 11 11 11

N12_M2/15, 12 11 11 11 11 11 11 11 11 11 11 11 11 11 11 11 12 11 11 11 11 11 11 11 11 11 11 11 11 11 11

N12_M2/16, 22 11 11 11 11 11 11 11 11 11 11 11 11 11 11 11 11 11 11 11 11 11 11 11 11 11 11 11 11 11 11

N12_M2/17, 12 11 11 11 11 11 11 11 11 11 11 11 11 11 11 11 12 11 11 11 11 11 11 11 11 11 11 11 11 11 11

N12_M2/18, 22 11 11 11 11 11 11 11 11 11 11 11 11 11 11 11 11 11 11 11 11 11 11 11 11 11 11 11 11 11 11

N12_M2/19, 22 11 11 11 11 11 11 11 11 11 11 11 11 11 11 11 12 11 11 11 11 11 11 11 11 11 11 11 11 11 11

N12_M2/20, 22 11 11 11 11 11 11 11 11 11 11 11 11 11 11 11 12 11 11 11 11 11 11 11 11 11 11 11 11 11 11

N12_M2/21, 22 11 11 11 11 11 11 11 11 11 11 11 11 11 11 11 12 11 11 11 11 11 11 11 11 11 11 11 11 11 11

N13_S1/01, 12 11 11 11 11 11 11 11 11 11 11 11 11 11 11 11 12 11 11 11 11 11 11 11 11 11 11 11 11 11 11

N13_S1/02, 12 11 11 11 11 11 11 11 11 11 11 11 11 11 11 11 12 11 11 11 11 11 11 11 11 11 11 11 11 11 11

N13_S1/03, 22 11 11 11 -9 11 11 11 11 11 11 11 11 11 11 11 12 11 11 11 11 11 11 11 11 11 11 11 11 11 11

N13_S1/04, 11 11 11 11 11 11 11 11 11 11 11 11 11 11 11 11 11 11 11 11 11 11 11 11 11 11 11 11 11 11 11

N13_S1/05, 12 11 11 11 11 11 11 11 11 11 11 11 11 11 11 11 11 11 11 11 11 11 11 11 11 11 11 11 11 11 11

N13_S1/06, 12 11 11 11 11 11 11 11 11 11 11 11 11 11 11 11 11 11 11 11 11 11 11 11 11 11 11 11 11 11 11

N13_S1/07, 12 11 11 11 11 11 11 11 11 11 11 11 11 11 11 11 12 11 11 11 11 11 11 11 11 11 11 11 11 11 11

N13_S1/08, 12 11 11 11 11 11 -9 11 11 11 11 11 11 11 11 11 12 11 11 11 11 11 11 11 11 11 11 11 11 11 11

N13_S1/09, 12 11 11 11 11 11 11 11 11 11 11 11 11 11 11 11 11 11 11 11 11 11 11 11 11 11 11 11 11 11 11

N13_S1/10, 12 11 11 11 11 11 11 11 11 11 11 11 11 11 11 11 11 11 11 11 11 11 11 11 11 11 11 11 11 11 11

N13_S1/11, 12 11 11 11 11 11 11 11 11 11 11 11 11 11 11 11 11 11 11 11 11 11 11 11 11 11 11 11 11 11 11

N13_S1/12, 22 11 11 11 11 11 11 11 11 11 11 11 11 11 11 11 11 11 11 11 11 11 11 11 11 11 11 11 11 11 11

N14_S3/01, 12 11 11 11 11 11 11 11 11 11 11 11 11 11 11 11 12 11 11 11 11 11 11 11 11 11 11 11 11 11 11

N14_S3/02, 12 11 11 11 11 11 11 11 11 11 11 11 11 11 11 11 11 11 11 11 11 11 11 11 11 11 11 11 11 11 11

N14_S3/03, 12 11 11 11 11 11 11 11 11 11 11 11 11 11 11 11 12 11 11 11 11 11 11 11 11 11 11 11 11 11 11

N14_S3/04, 22 11 11 11 11 11 11 11 11 11 11 11 11 11 11 11 12 11 11 11 11 11 11 11 11 11 11 11 11 11 11

N14_S3/05, 12 11 11 11 11 11 11 11 11 11 11 11 11 11 11 11 12 11 11 11 11 11 11 11 11 11 11 11 11 11 11

N14_S3/06, 12 11 11 11 11 11 11 11 11 11 11 11 11 11 11 11 12 11 11 11 11 11 11 11 11 11 11 11 11 11 11

N14_S3/07, 12 11 11 11 11 11 11 11 11 11 11 11 11 11 11 11 11 11 11 11 11 11 11 11 11 11 11 11 11 11 11

N14_S3/08, 12 11 11 11 11 11 11 11 11 -9 11 11 11 11 11 11 12 11 11 11 11 11 11 11 11 11 11 11 11 11 11

N14_S3/09, 12 11 11 11 11 11 11 11 11 11 11 11 11 11 11 11 12 11 11 11 11 11 11 11 11 11 11 11 11 11 11

N14_S3/10, 22 11 11 11 11 11 11 11 11 11 11 11 11 11 11 11 11 11 11 11 11 11 11 11 11 11 11 11 11 11 11

N14_S3/11, 12 11 11 11 11 11 11 11 11 11 11 11 11 11 11 11 12 11 11 11 11 11 11 11 11 11 11 11 11 11 11

N14_S3/12, 12 11 11 11 11 11 11 11 11 11 11 11 11 11 11 11 11 11 11 11 11 11 11 11 11 11 11 11 11 11 11

N14_S3/13, 12 11 11 11 11 11 11 11 11 11 11 11 11 11 11 11 12 11 11 11 11 11 11 11 11 11 11 11 11 11 11

N14_S3/14, 12 11 11 11 11 11 11 11 11 11 11 11 11 11 11 11 12 11 11 11 11 11 11 11 11 11 11 11 11 11 11

N14_S3/15, 22 11 11 11 11 11 11 11 11 11 11 11 11 11 11 11 22 11 11 11 11 11 11 11 11 11 11 11 11 11 11

N14_S3/16, 12 11 11 11 11 11 11 11 11 11 11 11 11 11 11 11 11 11 11 11 11 11 11 11 11 11 11 11 11 11 11

N14_S3/17, 12 11 11 11 11 11 11 11 11 -9 11 11 11 11 11 11 22 11 11 11 11 11 11 11 11 11 11 11 11 11 11

N14_S3/18, 12 11 11 11 11 11 11 11 11 11 11 11 11 11 11 11 12 11 11 11 11 11 11 11 11 11 11 11 11 11 11

N14_S3/19, 12 11 11 11 11 11 11 11 11 11 11 11 11 11 11 11 12 11 11 11 11 11 11 11 11 11 11 11 11 11 11

N14_S3/20, 12 11 11 11 11 11 11 11 11 11 11 11 11 11 11 11 12 11 11 11 11 11 11 11 11 11 11 11 11 11 11

N14_S3/21, 12 11 11 11 11 11 11 11 11 11 11 11 11 11 11 11 11 11 11 11 11 11 11 11 11 11 11 11 11 11 11

N15_S6/01, 12 11 11 11 11 11 11 11 11 11 11 11 11 11 11 11 12 11 11 11 11 11 11 11 11 11 11 11 11 11 11

N15_S6/02, 11 11 11 11 11 11 11 11 11 11 11 11 11 11 11 11 11 11 11 11 11 11 11 11 11 11 11 11 11 11 11

N15_S6/03, 22 11 11 11 11 11 11 11 11 11 11 11 11 11 11 11 12 11 11 11 11 11 11 11 11 11 11 11 11 11 11

N15_S6/04, 12 11 11 11 11 11 11 11 11 11 11 11 11 11 11 11 12 11 11 11 11 11 11 11 11 11 11 11 11 11 11

N15_S6/05, 22 11 11 11 11 11 11 11 11 11 11 11 11 11 11 11 12 11 11 11 11 11 11 11 11 11 11 11 11 11 11

N15_S6/06, 12 11 11 11 11 11 11 11 11 11 11 11 11 11 11 11 11 11 11 11 11 11 11 11 11 11 11 11 11 11 11

N15_S6/07, 22 11 11 11 11 11 11 11 11 11 11 11 11 11 11 11 11 11 11 11 11 11 11 11 11 11 11 11 11 11 11

N15_S6/08, 11 11 11 11 11 11 11 11 11 11 11 11 11 11 11 11 11 11 11 11 11 11 11 11 11 11 11 11 11 11 11

N15_S6/09, 22 11 11 11 11 11 11 11 11 11 11 11 11 11 11 11 12 11 11 11 11 11 11 11 11 11 11 11 11 11 11

N15_S6/10, 22 11 11 11 11 11 11 11 11 11 11 11 11 11 11 11 11 11 11 11 11 11 11 11 11 11 11 11 11 11 11

N15_S6/11, 22 11 11 11 11 11 11 11 11 11 11 11 11 11 11 11 12 11 11 11 11 11 11 11 11 11 11 11 11 11 11

N15_S6/12, 12 11 11 11 11 11 11 11 11 11 11 11 11 11 11 11 11 11 11 11 11 11 11 11 11 11 11 11 11 11 11

N15_S6/13, 11 11 11 11 11 11 11 11 11 11 11 11 11 11 11 11 11 11 11 11 11 11 11 11 11 11 11 11 11 11 11

N15_S6/14, 22 11 11 11 11 11 11 11 11 11 11 11 11 11 11 11 11 11 11 11 11 11 11 11 11 11 11 11 11 11 11

N15_S6/15, 22 11 11 11 11 11 11 11 11 11 11 11 11 11 11 11 12 11 11 11 11 11 11 11 11 11 11 11 11 11 11

N15_S6/16, 12 11 11 11 11 11 11 11 11 11 11 11 11 11 11 11 11 11 11 11 11 11 11 11 11 11 11 11 11 11 11

N15_S6/17, 22 11 11 11 11 11 11 11 11 11 11 11 11 11 11 11 12 11 11 11 11 11 11 11 11 11 11 11 11 11 11

N15_S6/18, 22 11 11 11 11 11 11 11 11 11 11 11 11 11 11 11 12 11 11 11 11 11 11 11 11 11 11 11 11 11 11

N15_S6/19, 22 11 11 11 11 11 11 11 11 11 11 11 11 11 11 11 11 11 11 11 11 11 11 11 11 11 11 11 11 11 11

N16_S8/01, 22 11 11 11 11 11 11 11 11 11 11 11 11 11 11 11 11 11 11 11 11 11 11 11 11 11 11 11 11 11 11

N16_S8/02, 22 11 11 11 11 11 11 11 11 11 11 11 11 11 11 11 11 11 11 11 11 11 11 11 11 11 11 11 -9 11 11

N16_S8/03, 12 11 11 11 11 11 11 11 11 11 11 11 11 11 11 11 11 11 11 11 11 11 11 11 11 11 11 11 -9 11 11

N16_S8/04, 12 11 11 11 11 11 11 11 11 11 11 11 11 11 11 11 11 11 11 11 11 11 11 11 11 11 11 11 -9 11 11

N16_S8/05, 12 11 11 11 11 11 11 11 11 11 11 11 11 11 11 11 11 11 11 11 11 11 11 11 11 11 11 11 -9 11 11

N16_S8/06, 12 11 11 11 11 11 11 11 11 11 11 11 11 11 11 11 11 11 11 11 11 11 11 11 11 11 11 11 -9 11 11

N16_S8/07, 12 11 11 11 11 11 11 11 11 11 11 11 11 11 11 11 11 11 11 11 11 11 11 11 11 11 11 11 -9 11 11

N16_S8/08, 22 11 11 11 11 11 11 11 11 11 11 11 11 11 11 11 11 11 11 11 11 11 11 11 11 11 11 11 -9 11 11

N16_S8/09, 12 11 11 11 11 11 11 11 11 11 11 11 11 11 11 11 11 11 11 11 11 11 11 11 11 11 11 11 -9 11 11

N16_S8/10, 22 11 11 11 11 11 11 11 11 11 11 11 11 11 11 11 11 11 11 11 11 11 11 11 11 11 11 11 -9 11 11

N16_S8/11, 22 11 11 11 11 11 11 11 11 11 11 11 11 11 11 11 11 11 11 11 11 11 11 11 11 11 11 11 -9 11 11

N16_S8/12, 22 11 11 11 11 11 11 11 11 11 11 11 11 11 11 11 11 11 11 11 11 11 11 11 11 11 11 11 -9 11 11

N16_S8/13, 22 11 11 11 11 11 11 11 11 11 11 11 11 11 11 11 11 11 11 11 11 11 11 11 11 11 11 11 -9 11 11

N16_S8/14, 22 11 11 11 11 11 11 11 11 11 11 11 11 11 11 11 11 11 11 11 11 11 11 11 11 11 11 11 11 11 11

N16_S8/15, 22 11 11 11 11 11 11 11 11 11 11 11 11 11 11 11 11 11 11 11 11 11 11 11 11 11 11 11 11 11 11

N16_S8/16, 11 11 11 11 11 11 11 11 11 11 11 11 11 11 11 11 11 11 11 11 11 11 11 11 11 11 11 11 11 11 11

N16_S8/17, 12 11 11 11 11 11 11 11 11 11 11 11 11 11 -9 11 11 11 11 11 11 11 11 11 11 11 11 11 11 11 11

N16_S8/18, 22 11 11 11 11 11 11 11 11 11 11 11 11 11 11 11 11 11 11 11 11 11 11 11 11 11 11 11 11 11 11

N16_S8/19, 11 11 11 11 11 22 11 11 11 11 11 11 11 11 11 11 11 11 11 11 11 11 11 11 11 11 11 11 11 11 11

N16_S8/20, 22 11 11 11 11 11 11 11 11 11 11 11 11 11 11 11 11 11 11 11 11 11 11 11 11 11 11 11 11 11 11

N16_S8/21, 22 11 11 11 11 11 11 11 11 11 11 11 11 11 11 11 11 11 11 11 11 11 11 11 11 11 11 11 11 11 11

N17_S10/02, 22 11 11 11 11 11 11 11 11 11 11 11 -9 11 11 11 11 11 11 11 11 11 11 11 11 11 11 11 -9 11 11

N17_S10/03, 12 11 11 11 11 11 11 11 11 11 11 11 11 11 11 11 11 11 11 11 11 11 11 11 11 11 11 11 -9 11 11

N17_S10/04, 11 11 11 11 11 11 11 11 11 11 11 11 11 11 11 11 11 11 11 11 11 11 11 11 11 11 11 11 11 11 11

N17_S10/05, 22 11 11 11 11 11 11 11 11 11 11 11 11 11 11 11 12 11 11 11 11 11 11 11 11 11 11 11 11 11 11

N17_S10/06, 12 11 11 11 11 11 11 11 11 11 11 11 -9 11 11 11 11 11 11 -9 11 11 11 -9 -9 11 11 11 -9 11 11

N17_S10/07, 22 11 11 11 11 11 11 11 11 11 11 11 -9 11 11 11 11 11 11 11 11 11 11 11 11 11 11 11 -9 11 11

N17_S10/08, 22 11 11 11 11 11 11 11 11 11 11 11 11 11 11 11 11 11 11 11 11 11 11 11 11 11 11 11 -9 11 11

N17_S10/09, 12 11 11 11 11 11 11 11 11 11 11 11 11 11 11 11 12 11 11 11 11 11 11 11 11 11 11 11 -9 11 11

N17_S10/10, 22 -9 11 11 11 11 11 11 11 -9 11 11 -9 11 11 11 12 11 11 -9 11 11 11 -9 11 11 11 11 -9 11 11

N17_S10/11, 11 11 11 11 11 11 11 11 11 11 11 11 -9 11 11 11 12 11 11 11 11 11 11 11 11 11 11 11 -9 11 11

N17_S10/13, 12 11 11 11 11 11 11 11 11 11 11 11 11 11 11 11 11 -9 11 11 11 11 11 11 11 11 -9 11 -9 11 11

N17_S10/15, 22 11 11 11 11 11 11 11 11 11 11 11 11 11 11 11 11 11 11 11 11 11 11 11 11 11 11 11 -9 11 11

N17_S10/16, 12 11 11 11 11 11 11 11 11 11 11 11 -9 11 11 11 11 11 11 11 11 11 11 11 11 11 11 11 -9 11 11

N17_S10/17, 12 11 11 11 11 11 11 11 11 11 11 11 11 11 11 11 12 11 11 11 11 11 11 11 11 11 11 11 11 11 11

N17_S10/18, 12 11 11 11 11 11 11 11 11 11 11 11 -9 11 11 11 12 11 11 11 11 11 11 11 11 11 11 11 -9 11 11

N17_S10/21, 11 11 11 11 11 11 11 11 11 11 11 11 -9 11 11 11 11 11 11 11 11 11 11 11 11 11 11 11 -9 11 11

N17_S10/22, -9 11 11 11 11 11 11 11 11 11 11 11 -9 11 11 11 -9 11 11 11 11 11 11 -9 11 11 11 11 -9 11 11

N17_S10/23, -9 11 11 11 11 11 11 11 11 -9 11 11 -9 11 11 11 -9 11 11 -9 11 11 11 -9 11 11 11 11 -9 11 11

N18_S12/01, 12 11 11 11 11 11 11 11 11 11 11 11 -9 11 11 11 11 11 11 11 11 11 11 11 11 11 11 11 -9 11 11

N18_S12/02, 11 11 11 11 11 11 11 11 11 11 11 11 -9 11 11 11 11 11 11 11 11 11 11 -9 11 -9 11 11 -9 11 11

N18_S12/03, 12 11 11 11 11 11 11 11 11 11 11 11 -9 11 11 11 11 11 11 11 11 11 11 11 11 11 11 11 -9 11 11

N18_S12/04, 22 11 11 11 11 11 11 11 11 11 11 11 -9 11 11 11 11 11 11 11 11 11 11 -9 11 11 11 11 -9 11 11

N18_S12/05, -9 -9 11 11 11 -9 11 11 11 11 11 11 -9 11 11 11 11 11 11 11 11 11 11 -9 -9 11 11 11 -9 -9 11

N18_S12/06, 22 11 11 11 11 11 11 11 11 11 11 11 -9 11 11 -9 11 11 11 11 11 11 11 -9 11 11 11 11 -9 11 11

N18_S12/07, 22 11 11 11 11 11 11 11 11 11 11 11 -9 11 11 11 12 11 11 11 11 11 11 -9 11 11 11 11 -9 11 11

N18_S12/08, 22 11 11 11 11 11 -9 11 11 11 11 11 -9 11 11 11 11 11 11 11 11 11 11 -9 11 11 11 11 -9 11 11

N18_S12/09, 22 11 11 11 11 11 11 11 11 11 11 11 -9 11 11 11 12 11 11 11 11 11 11 -9 11 11 11 11 -9 11 11

N18_S12/10, 11 11 11 11 11 11 11 11 11 11 11 11 -9 11 11 11 11 11 11 11 11 11 11 11 11 11 11 11 -9 11 11

N18_S12/11, 12 11 11 11 11 11 11 11 11 11 11 11 -9 11 11 11 12 11 11 11 11 11 11 11 11 11 11 11 -9 11 11

N18_S12/12, 22 11 11 11 11 11 11 11 11 11 11 11 -9 11 11 11 11 11 11 11 11 11 11 11 11 11 11 11 -9 11 11

N18_S12/13, 22 11 11 11 11 11 11 11 11 11 11 11 -9 11 11 11 11 11 11 11 11 11 11 11 11 11 11 11 -9 11 11

N18_S12/14, 12 11 11 11 11 11 11 11 11 11 11 11 -9 11 11 11 11 11 11 11 11 11 11 11 11 11 11 11 -9 11 11

N18_S12/15, 12 11 11 11 11 11 11 11 11 11 11 11 -9 11 11 11 11 11 11 11 11 11 11 11 11 11 11 11 -9 -9 11

N18_S12/16, 22 11 11 11 11 11 11 11 11 11 11 11 -9 11 11 11 11 11 11 11 11 11 11 11 11 11 11 11 -9 11 11

N18_S12/17, 22 11 11 11 11 11 11 11 11 11 11 11 -9 11 11 11 12 11 11 11 11 -9 11 11 11 11 11 11 -9 11 11

N18_S12/18, 22 11 11 11 11 11 11 11 11 11 11 11 -9 11 11 11 22 11 11 11 11 11 11 11 11 11 11 11 -9 11 11

N18_S12/19, 12 11 11 11 11 -9 11 11 11 11 11 11 -9 11 11 11 11 11 11 11 11 11 11 11 11 11 11 11 -9 11 11

N18_S12/20, 12 11 11 11 11 11 11 11 11 11 11 11 11 11 11 11 12 11 11 11 11 11 11 11 11 11 11 11 11 11 11

N18_S12/21, 22 11 11 11 11 11 11 11 11 11 11 11 -9 11 11 11 11 11 11 11 11 11 11 11 11 11 11 11 -9 11 11

N19_T1/01, 22 11 11 11 11 11 11 11 11 11 11 11 11 11 11 11 12 11 11 11 11 11 11 11 11 11 11 11 11 11 11

N19_T1/02, 22 11 11 11 11 11 11 11 11 11 11 11 11 11 11 11 12 11 11 11 11 11 11 11 11 11 11 11 11 11 11

N20_T2/01, 12 11 11 11 11 11 11 11 11 11 11 11 11 11 11 11 12 11 11 11 11 11 11 11 11 11 11 11 11 11 11

N20_T2/02, 22 11 11 11 11 11 11 11 11 -9 11 11 11 11 11 11 12 11 11 11 11 11 11 11 11 11 11 11 11 11 11

N20_T2/03, 22 11 11 11 11 11 11 11 11 11 11 11 11 11 11 11 12 11 11 11 11 11 11 11 11 11 11 11 11 11 11

N20_T2/04, 22 11 11 11 11 11 11 11 11 11 11 11 11 11 11 11 -9 11 11 11 11 11 11 11 11 11 11 11 11 11 11

N20_T2/05, 22 11 11 11 11 11 11 11 11 11 11 11 11 11 11 11 12 11 11 11 11 11 11 11 11 11 11 11 11 11 11

N20_T2/06, 22 11 11 11 11 11 11 11 11 11 11 11 11 11 11 11 12 11 11 11 11 11 11 11 11 11 11 11 11 11 11

N20_T2/07, 22 11 11 11 11 11 11 11 11 11 11 11 11 11 11 11 12 11 11 11 11 11 11 11 11 11 11 11 11 11 11

N20_T2/08, 22 11 11 11 11 11 11 11 11 11 11 11 11 11 11 11 12 11 11 11 11 11 11 11 11 11 11 11 11 11 11

N20_T2/09, 12 11 11 11 11 11 11 11 11 11 11 11 11 11 11 11 11 11 11 11 11 11 11 11 11 11 11 11 11 11 11

N20_T2/10, 22 11 11 11 11 11 11 11 11 11 11 11 11 11 11 11 12 11 11 11 11 11 11 11 11 11 11 11 11 11 11

N20_T2/11, 12 11 11 11 11 11 11 11 11 11 11 11 11 11 11 11 11 11 11 11 11 11 11 11 11 11 11 11 11 11 11

N20_T2/12, 22 11 11 11 11 11 11 11 11 11 11 11 11 11 11 11 12 11 11 11 11 11 11 11 11 11 11 11 11 11 11

N20_T2/13, 22 11 11 11 11 11 11 11 11 11 11 11 11 11 11 11 11 11 11 11 11 11 11 11 -9 11 11 11 11 11 11

N20_T2/14, 22 11 11 11 11 11 11 11 11 11 11 11 11 11 11 11 12 11 11 11 11 11 11 11 11 11 11 11 11 11 11

N20_T2/15, 22 11 11 11 11 11 11 11 11 11 11 11 11 11 11 11 12 11 11 11 11 11 11 11 11 11 11 11 11 11 11

N20_T2/16, 22 11 11 11 11 11 11 11 11 11 11 11 11 11 11 11 12 11 11 11 11 11 11 11 11 11 11 11 11 11 11

N20_T2/17, 11 11 11 11 11 11 11 11 11 11 11 11 11 11 11 11 11 11 11 11 11 11 11 11 11 11 11 11 11 11 11

N20_T2/18, 12 11 11 11 11 11 11 11 11 11 11 11 11 11 11 11 11 11 11 11 11 11 11 11 11 11 11 11 11 11 11

N20_T2/19, 22 11 11 11 11 11 11 11 11 11 11 11 11 11 11 11 11 11 11 11 11 11 11 11 11 11 11 11 11 11 11

N20_T2/20, 22 11 11 11 11 11 11 11 11 11 11 11 11 11 11 11 12 11 11 11 11 11 11 11 11 11 11 11 11 11 11

N20_T2/21, 22 11 11 11 11 11 11 11 11 11 11 11 11 11 11 11 12 11 11 11 11 11 11 11 11 11 11 11 11 11 11

N20_T2/22, 22 11 11 11 11 11 11 11 11 11 11 11 11 11 11 11 12 11 11 11 11 11 11 11 11 11 11 11 11 11 11

N20_T2/23, 22 11 11 11 11 11 11 11 11 11 11 11 11 11 11 11 12 11 11 11 11 11 11 11 11 11 11 11 11 11 11

N20_T2/24, 22 11 11 11 11 11 11 11 11 11 11 11 -9 11 11 11 11 11 11 11 11 11 11 11 11 11 11 11 11 11 11

N20_T2/25, 22 11 11 11 11 11 11 11 11 11 11 11 11 11 11 11 11 11 11 11 11 11 11 11 11 11 11 11 11 11 11

N20_T2/26, 22 11 11 11 11 11 11 11 11 11 11 11 11 11 11 11 11 11 11 11 11 11 11 11 11 11 11 11 11 11 11

N21_T3/01, 22 11 11 11 11 11 11 11 11 11 11 11 11 11 11 11 11 11 11 11 11 11 11 11 11 11 11 11 11 11 11

N21_T3/02, 12 11 11 11 11 11 11 11 11 11 11 11 11 11 11 11 11 11 11 11 11 11 11 11 11 11 11 11 11 11 11

N21_T3/03, 12 11 11 11 11 11 11 11 11 11 11 11 11 11 11 11 11 11 11 11 11 11 11 11 11 11 11 11 11 11 11

N21_T3/04, 22 11 11 11 11 11 11 11 11 11 11 11 11 11 11 11 11 11 11 11 11 11 11 11 11 11 11 11 11 11 11
